# Flanking‐Group Engineering Unlocks Emerging Functionalities in Diketopyrrolopyrrole Semiconductors

**DOI:** 10.1002/advs.76548

**Published:** 2026-07-23

**Authors:** Xiaoyun Wu, Shanshan Zhou, Meng Li, Aung Ko Ko Kyaw, Xiao‐Lei Shi, Qian Liu

**Affiliations:** ^1^ School of Chemistry and Physics ARC Research Hub in Zero‐emission Power Generation for Carbon Neutrality, and Centre for Materials Science Queensland University of Technology Brisbane Queensland Australia; ^2^ Department of Electronic and Electrical Engineering Southern University of Science and Technology Shenzhen China

**Keywords:** electronics, emerging technologies, material system, nanotechnology, nitrile, organic semiconductor, stretchable transistor

## Abstract

Diketopyrrolopyrrole (DPP) is a cornerstone building block in high‐performance organic semiconductors, valued for its strong π‐conjugation, chemical robustness, and tunable optoelectronic characteristics. Conventional DPP synthesis relies on aromatic nitrile precursors, establishing a design paradigm in which flanking aromatic units govern energy levels, molecular packing, and charge transport. Although systematic variation of these flanking groups has enabled substantial performance gains, this strategy inherently constrains structural diversity and limits functional adaptability, particularly for emerging technologies requiring flexibility, biointegration, and multimodal responsiveness. Recent advances move beyond this conventional framework by reengineering DPP flanking architectures and molecular topologies. In addition to enabling new functionalities in established material systems, unconventional design strategies, such as naphthalene‐based motifs, vinyl linkages, and fused DPP backbones, have been developed to reshape electronic coupling and intermolecular interactions. These approaches expand DPP functionality beyond traditional transistor and photovoltaic roles toward flexible electronics, intelligent skins, and (bio)sensing platforms. By establishing flanking‐group engineering as a unifying design paradigm, this review provides a coherent structure‐property‐application framework for DPP‐based semiconductors, offering generalizable guidelines for the development of next‐generation multifunctional organic electronics.

## Graphical abstract

This review presents a comprehensive and unified perspective of flanking‐group engineering in Diketopyrrolopyrrole (DPP) based organic semiconductors that connects molecular‐level design with cross‐device functionalities. By integrating insights from molecular design, solid‐state packing, and device physics, this review seeks to establish general design principles that enable the development of next‐generation DPP materials for multifunctional and integrated electronic systems.

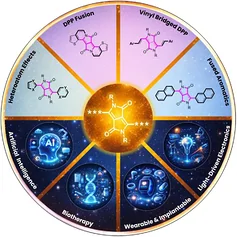

## 1 Introduction

Organic semiconductors (OSCs) have emerged as a pivotal class of functional materials for next‐generation (opto)electronic technologies, owing to their intrinsic advantages, such as mechanical flexibility, solution processability, structural and chemical tunability, and potential biocompatibility [1, 2, 3, 4, 5, 6, 7, 8, 9, 10, 11, 12]. These attributes enable large‐area, printed manufacturing, and seamless integration with flexible, stretchable, and biological substrates, thereby unlocking diverse application scenarios that are challenging or inaccessible for conventional rigid inorganic materials. Based on their fundamental π‐conjugated building blocks, such as thiophene, diketopyrrolopyrrole (DPP), benzodithiophene, and other motifs, OSCs can be systematically classified into distinct material families. However, the performance requirements imposed on active OSCs are highly device‐specific and often vary substantially, or even conflict, across different applications [13, 14, 15, 16, 17, 18, 19, 20]. Consequently, OSCs derived from a single building block are typically optimized for a narrow range of device platforms, which restrict both the accessible molecular design space and the breadth of practical applications.

Against this backdrop, DPP has distinguished itself as a particularly versatile building block for constructing OSCs across diverse electronic and optoelectronic devices [13]. The DPP motif features a fused bicyclic lactam core with strong electron‐withdrawing character, excellent chemical robustness, and outstanding photochemical stability. These structural characteristics impart DPP‐based OSCs with a unique combination of tunable electronic and optical properties. Organic field‐effect transistors (OFETs) and organic photovoltaics (OPVs), where DPP has been extensively explored since its discovery, provide representative examples. In OFETs, the pronounced electron deficiency of DPP facilitates the design of diverse donor–acceptor (D–A) and acceptor–acceptor (A–A) conjugated systems, enabling precise modulation of frontier molecular orbitals for high‐performance p‐type, n‐type, and ambipolar semiconductors [21]. Moreover, the rigid and planar DPP backbone, together with its hydrogen‐bonding sites, promotes dense molecular packing and long‐range order in the solid state, leading to charge carrier mobilities exceeding 10 cm^2^ V^−1^ s^−1^ [22]. In OPVs, the planar conjugated backbone of DPP enhances π‐electron delocalization and intramolecular charge transfer within D–A architectures, resulting in high molar extinction coefficients and broad absorption extending from the visible to near‐infrared (NIR) region, the features that are highly desirable for efficient solar light harvesting. Furthermore, the energy levels of DPP‐based OSCs can be finely tuned through molecular engineering, enabling favorable energetic alignment with both fullerene and nonfullerene acceptors (NFAs) [23]. Beyond OFETs and OPVs, DPP‐based OSCs have also been explored in thermoelectric devices, photodetectors, and light‐emitting devices (LEDs), underscoring their broad applicability across multiple device platforms [24, 25, 26, 27, 28, 29].

**FIGURE 1:**
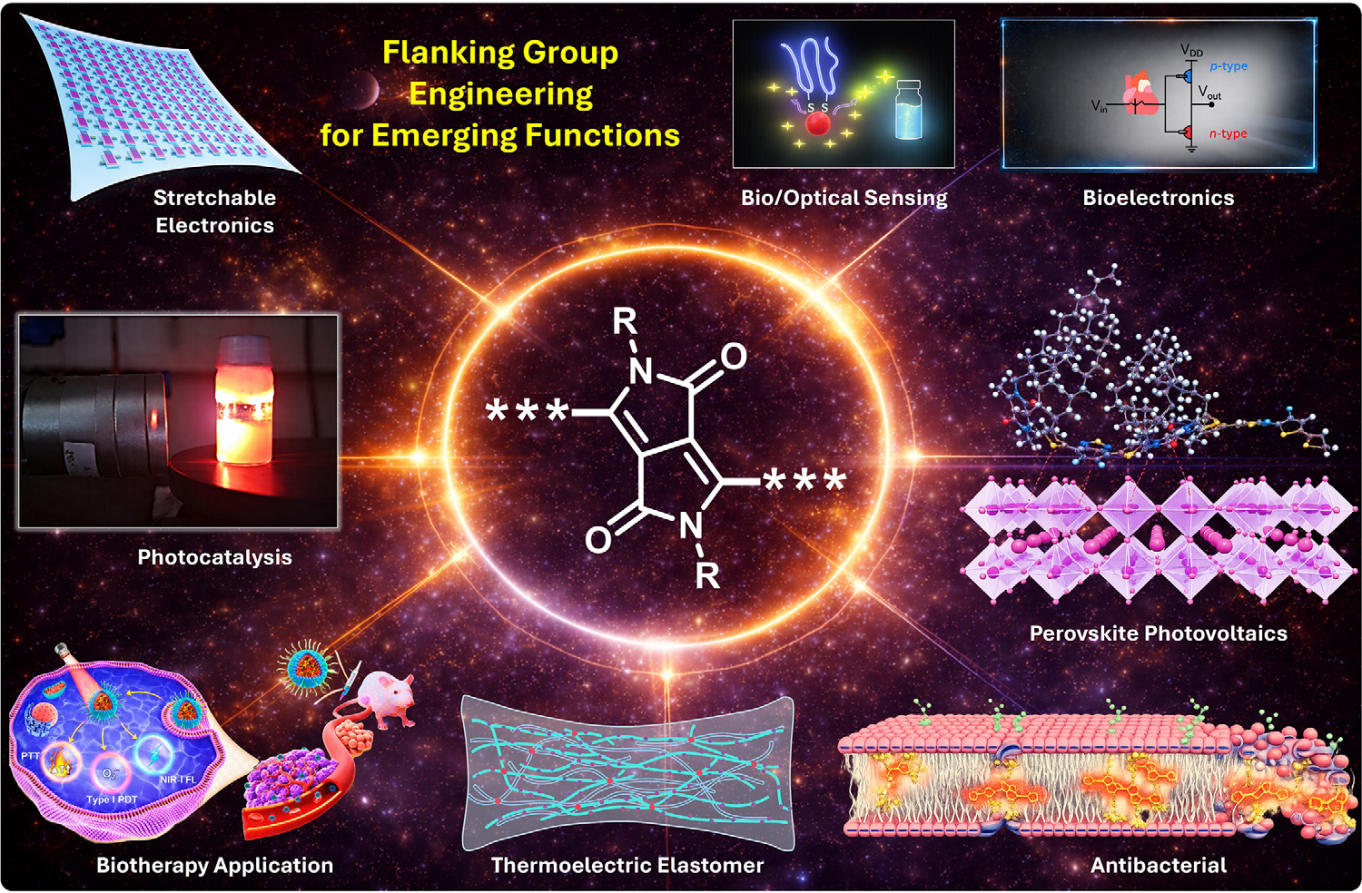
Schematic overview of emerging functionalities in diketopyrrolopyrrole (DPP) based materials enabled by flanking‐group engineering.

Despite their exceptional versatility, extending DPP‐based OSCs to frontier applications (Figure 1), such as wearable and implantable electronics, biointegrated sensors, and multifunctional soft devices, remains a significant challenge. In contrast to conventional electronic platforms, these emerging applications impose requirements that extend well beyond high electronic performance, encompassing mechanical compliance, environmental stability, and biocompatibility [30, 31, 32, 33, 34, 35, 36, 37, 38]. In this context, the molecular design of flanking groups, which defines and diversifies DPP‐based material families, plays a decisive role.

Flanking‐group engineering in DPP‐based semiconductors has been extensively explored and systematically reviewed, particularly in the context of optimizing charge transport properties for organic field‐effect transistors and photovoltaic devices. However, most existing studies and reviews primarily focus on device‐specific performance metrics or electronic structure modulation, with relatively limited attention to how flanking‐group design can be leveraged to simultaneously address multifunctional requirements across emerging device platforms, especially those involving coupled electronic‐ionic transport, mechanical adaptability, and bio‐interfacing characteristics.

In this regard, a comprehensive and unified perspective that connects molecular‐level design with cross‐device functionalities remains largely underdeveloped. In particular, how flanking‐group engineering can be utilized to bridge traditionally separate application domains, such as transistors, thermoelectrics, and bioelectronics, and to reconcile competing property requirements (e.g., charge transport vs. ionic interaction, rigidity vs. flexibility) is not yet systematically articulated.

This review aims to fill this gap by shifting the focus from conventional device‐oriented optimization toward a function‐oriented and cross‐platform design paradigm. We summarize recent advances in DPP‐based OSCs for emerging applications since 2020, emphasizing how flanking‐group engineering governs structure‐property‐function relationships across diverse device architectures. By integrating insights from molecular design, solid‐state organization, and device physics, this review seeks to establish general design principles that enable the development of next‐generation DPP materials for multifunctional and integrated electronic systems.

## 2 Five‐Membered Aromatic Flanking of DPP

Aromatic thiophene has been the most extensively studied flanking group for DPP since the early stage of DPP research for OFETs and OPVs, owing in part to favorable hydrogen‐bonding interactions between the thiophene units and the DPP core. Building on this foundation, other five‐membered aromatic heterocycles, including furan, selenophene, and thiazole, have subsequently introduced as alternative flanking units to further diversify DPP‐based molecular architectures. Among these systems, thiophene‐flanked DPP (DPPT) derivatives remain the most widely investigated and therefore prioritized in the exploration of emerging applications, benefiting from the deep understanding of their structure–property relationships established through an extensive body of prior studies [39, 40, 41, 42, 43, 44, 45, 46, 47, 48, 49, 50, 51, 52, 53, 54, 55, 56, 57, 58, 59, 60, 61, 62, 63, 64, 65, 66, 67, 68, 69, 70, 71, 72, 73, 74, 75, 76, 77, 78, 79, 80, 81, 82, 83, 84, 85, 86, 87, 88, 89, 90, 91, 92, 93, 94, 95, 96, 97, 98].

### 2.1 Design Strategies for Stretchable Electronics Based on Thiophene‐Flanked DPP

**FIGURE 2:**
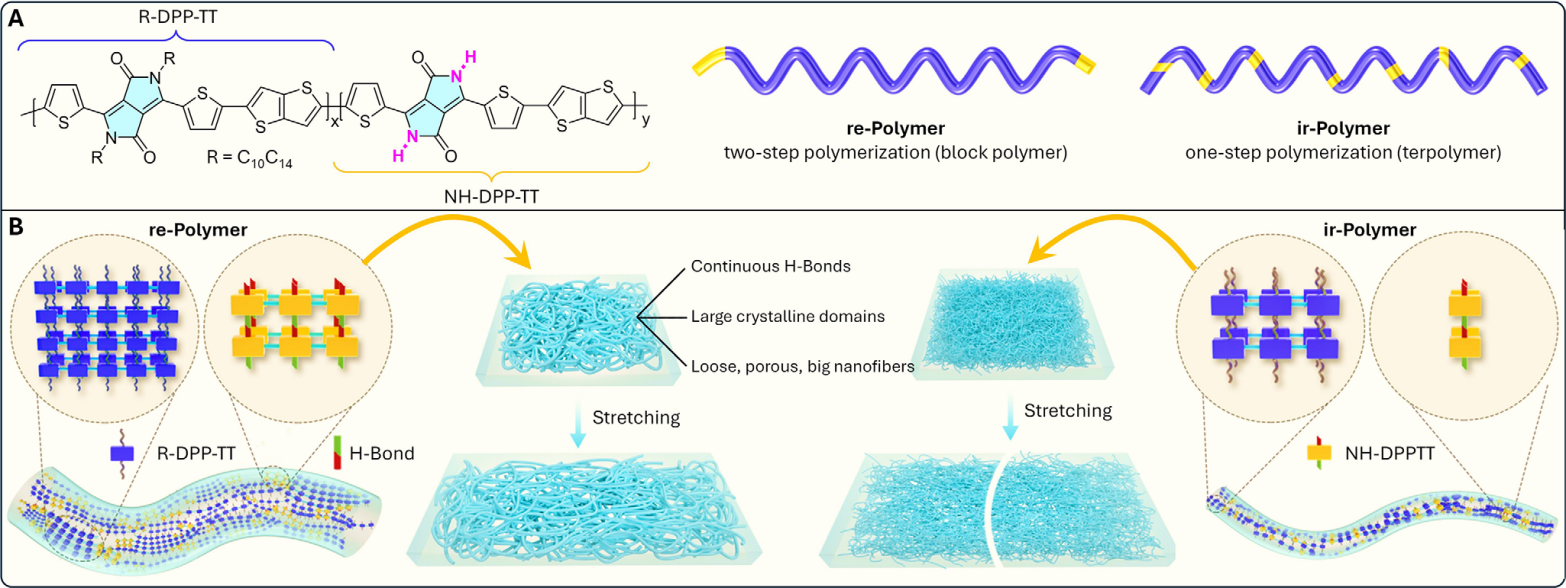
(A) Chemical structures and backbone schematics of DPP‐TT based re‐polymer and ir‐polymer, and (B) the comparison of their stretchability mechanisms. Adapted with permission [113]. Copyright 2024, AAAS.

As the most extensively studied flanking unit, thiophene not only defines the electronic structure and backbone planarity of DPP semiconductors but also establishes a structural foundation upon which various secondary engineering strategies are built, including side‐chain modification, copolymer design, and device integration. One major area in which DPPT‐based materials have demonstrated significant impact is flexible and stretchable electronics, which are critically important for emerging skin‐like and wearable applications. In this context, the Bao group pioneered a molecular design strategy that balances polymer film crystallinity with mechanical stretchability, enabling the simultaneous realization of high electronic performance and mechanical compliance. This concept has since been successfully extended to a wide range of molecular systems [99, 100, 101, 102]. A persistent challenge in such designs is mitigating the mobility loss associated with reduced crystallinity. This issue is particularly pronounced in high‐mobility polymers such as DPP‐TT (a copolymer comprising thiophene‐flanked DPP and thieno[3,2‐*b*]thiophene), which exhibits hole mobilities exceeding 10 cm^2^ V^−1^ s^−1^ [22], and has consequently emerged as a model system for investigating emerging functionalities [103, 104, 105, 106, 107, 108, 109, 110, 111, 112]. To address this issue, Yue et al. introduced strong hydrogen bonding into the DPP‐TT polymer backbone by incorporating thermally removable 2‐ethylhexoxycarbonyl side chains, which expose NH groups (Figure 2A) upon annealing [113]. The resulting intermolecular hydrogen‐bonding interactions markedly enhanced the intrinsic stretchability of the polymer, while the thiophene flanking groups, through intramolecular hydrogen bonding with the DPP core and noncovalent interactions with TT unit, promote a highly planar backbone, thereby preserving its high crystallinity. Importantly, this study further demonstrated that the re‐polymer, a block copolymer synthesized via a two‐step process, exhibits substantially higher stretchability than the ir‐polymer, a terpolymer synthesized via a one‐step process. This difference originates from their distinct nanostructures (Figure 2B) that, compared with ir‐polymer, the re‐polymer features larger crystalline domains, more continuous hydrogen‐bonding networks, and looser, porous nanofibers with large dimensions. These structural characteristics collectively enable the polymer to simultaneously maintain high stretchability and high charge transport performance, yielding hole mobilities exceeding 1.0 cm^2^ V^−1^ s^−1^ even at 100% strain. This work therefore provides an effective molecular and structural design strategy for simultaneously achieving high crystallinity and high stretchability in DPP‐based semiconductors.

**FIGURE 3:**
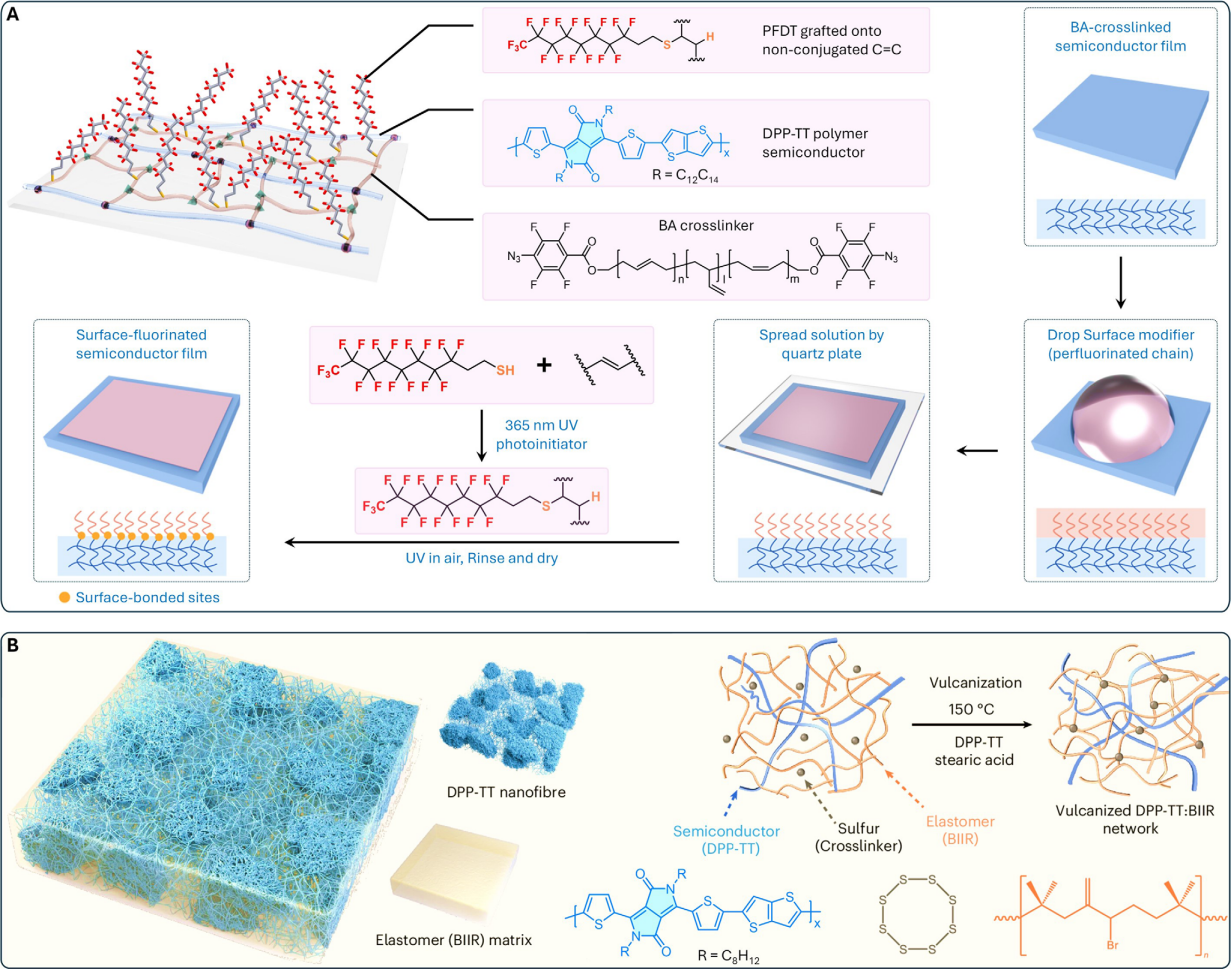
(A) Schematic illustration and fabrication procedure of fluorination‐protected stretchable films. Adapted with permission [115]. Copyright 2023, Nature. (B) Sulfur‐crosslinked biocompatible elastomer film incorporating DPP‐TT for implantable applications. Adapted with permission [4]. Copyright 2025, Nature.

Another effective strategy for enhancing stretchability in DPP‐based OSCs involves the deliberate introduction of crosslinking sites to construct mechanically robust polymer networks. Under this strategy, benefiting from the thiophene flanking groups for highly planar DPP‐TT backbone, the induced high crystallinity and tight molecular packing preserve its intrinsic aggregation, even upon blending with a third component or undergoing crosslinking reactions to impart stretchability. Consequently, continuous charge‐transport pathways are retained, enabling efficient carrier transport under applied strain. Zheng et al. developed a covalently embedded in situ rubber matrix (i‐RUM) strategy, in which i‐RUM precursors are initially mixed with both the substrate and the semiconducting polymer, followed by azide‐based cross‐linking to form high‐density covalent networks that substantially improve stretchability [114]. This approach was further advanced through the incorporation of a photoinitiated fluorinated compound to enhance the environmental operational stability [115]. As illustrated in Figure 3A, the high fluorine density forms a tightly packed nanostructure that serves as an effective barrier against moisture ingress and photoinduced degradation, while remaining covalently bonded to the active layer during stretching. Consequently, fluorinated devices retained hole mobilities of approximately 1.0 cm^2^ V^−1^ s^−1^ under high humidity, whereas nonfluorinated devices suffered a dramatic mobility drop to 10^−6^ cm^2^ V^−1^ s^−1^. Complementary to covalent network engineering, Jung et al. introduced a biocompatible elastomer, bromo‐isobutyl‐isoprene rubber (BIIR), blended with DPP‐TT to form a sulfur‐cross‐linked nanocomposite (Figure 3B), thereby achieving both high stretchability and excellent biocompatibility. Notably, the composite did not impair cell viability, proliferation, or migration, nor did it trigger inflammation, while additionally exhibiting antibacterial properties. Subcutaneous implantation studies in BALB/c mice showed no significant inflammatory response or severe fibrous capsule formation, confirming suitability of this material system for long‐term implantation. Building on these favorable material properties, skin‐like implantable transistors and logic circuits, including active‐matrix arrays, inverters, NOR gates, and NAND gates, maintained stable operation under mechanical deformation and physiological conditions [4]. These results are particularly encouraging, as they demonstrate the simultaneous realization of mechanical robustness and practical biomedical applicability. Beyond material and device stability, the suppression of signal artifacts and drift induced by mechanical deformation remains a critical challenge for stretchable electronics in end‐user applications. To address this issue, Zhao et al. integrated DPP‐TT‐based field‐effect transistors with stretchable diodes to construct an integrated biosensor platform that reduced signal distortion by up to two orders of magnitude. This improvement was primarily attributed to capacitive coupling effects and the effective subtraction of interference signals using two extended gates independently functionalized with target and reference bioreceptors [3].

### 2.2 Molecular Engineering Toward Intrinsically Stretchable Polymers

**FIGURE 4:**
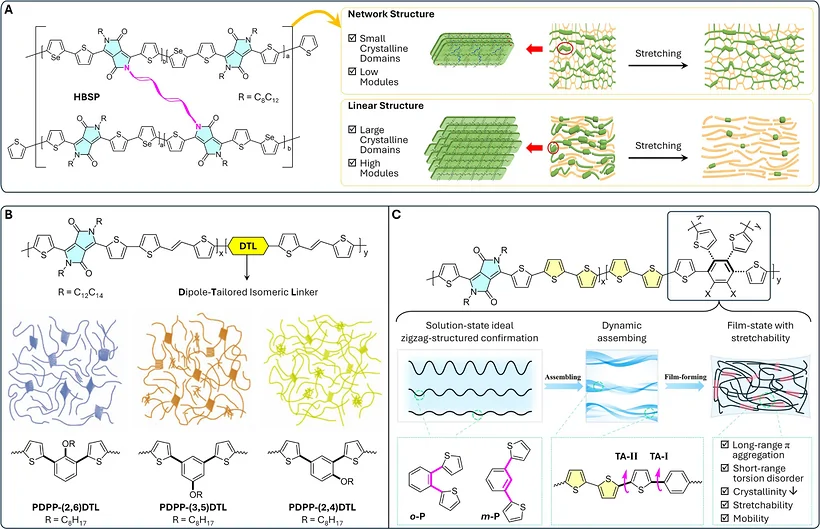
(A) Comparison of stretchability between pre‐endcapping polymer and its linear counterpart. Adapted with permission [116]. Copyright 2024, Wiley. (B) Stretchability enabled by a dipole‐tailored isomeric linker induced morphology modulation. Adapted with permission [117]. Copyright 2025, ACS. (C) Molecular design of zigzag polymers and the corresponding stretchability mechanism. Adapted with permission [118]. Copyright 2024, ACS.

Benefiting from the well‐established electronic structure and favorable backbone planarity imparted by the thiophene flanking units, DPPT‐based polymers provide a robust platform for further molecular engineering toward intrinsic stretchability. At the molecular design level, early approaches introduced flexible spacers into the polymer backbones to balance stretchability and electrical performance. However, such modifications often disrupted backbone conjugation and severely compromised charge transport, limiting their widespread application in flexible and stretchable electronics. To overcome this limitation, Zhou et al. proposed a side‐chain‐directed backbone‐linking strategy, as illustrated in Figure 4A, in which polymer backbones are interconnected through the flexible alkyl side chains rather than along the conjugated main chain. This design preserves the structural integrity of the conjugated backbone for efficient charge transport, while reducing crystalline domain size and elastic modulus, both of which remain relatively unchanged under strain. Consequently, the resulting polymers exhibit simultaneous improvement in both charge transport and stretchability compared with analogous single‐chain polymers lacking side‐chain linkages [116].

Beyond connectivity engineering, Kang and Zhu et al. systematically investigated the role of backbone geometry and conformational distorting in governing stretchability. Using a geometric molecular design strategy, Kang et al. demonstrated that the molecular dipole moment plays a critical role in regulating the trade‐off between crystallinity, aggregation and charge transport. Through isomeric linker engineering (Figure 4B), PDPP‐(2,4)DTL exhibited a higher dipole moment than PDPP‐(2,6)DTL and PDPP‐(3,5)DTL. This large dipole moment suppressed long‐range crystallinity while promoting short‐range aggregation, thereby maintaining continuous pathways even under 80% strain. Consequently, when blended with Y7, the corresponding photodiode retained 73% of its external quantum efficiency, with only a minor reduction in detectivity (∼10^11^ Jones) [117]. Complementarily, Zhu et al. reported that introducing a zigzag backbone geometry can endow polymer semiconductors with intrinsic stretchability without sacrificing electronic performance (Figure 4C). This structural design effectively decouples mechanical flexibility from charge transport by regulating backbone conformation and side‐chain‐induced crystallinity. As a result, charge carrier mobility was largely preserved under extreme deformation, decreasing only from 1.92 to 1.43 and 1.37 cm^2^ V^−1^ s^−1^ when stretched parallel and perpendicular to the charge transport direction, respectively, even at 100% strain [118].

**FIGURE 5:**
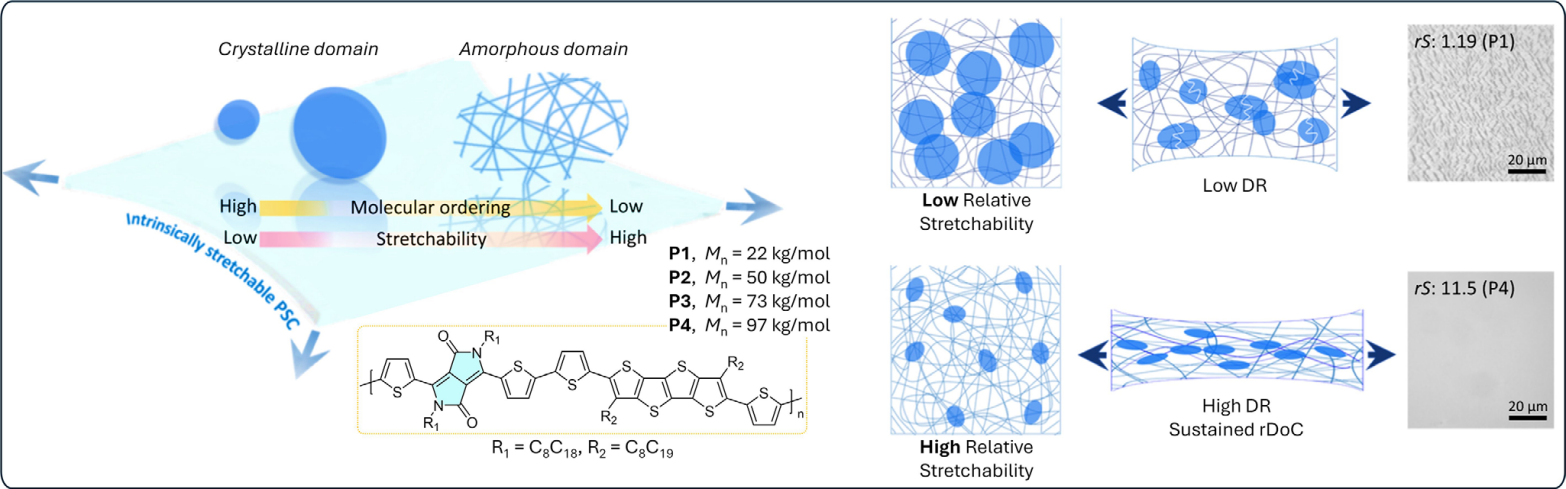
Influence of molecular weight on stretchability, and definition of relative stretchability (rS = *∆*DR/*∆*rDoC). DR: dichroic ratio, rDoC: relative degree of crystallinity. Adapted with permission [119]. Copyright 2023, Nature.

For polymer semiconductors, molecular weight is a critical parameter that intrinsically influences both charge transport and mechanical behavior. Wu et al. systematically investigated the effect of molecular weight on stretchability and demonstrated that ultrathin polymer semiconductor films (<100 nm) can simultaneously achieve high stretchability and stable charge transport through multimodal energy dissipation under mechanical strain. To quantify this behavior, they introduced a relative stretchability (rS) parameter, defined as the ratio of changes in polymer chain alignment (expressed by dichroic ratio, DR) to changes in the relative degree of crystallinity (rDoC). As shown in Figure 5, low‐molecular‐weight films with larger crystallites primarily accommodated strain through crystallite rotation, exhibited limited strain‐induced crystallization, and ultimately underwent amorphization and cracking due to stress localization. In contrast, high‐molecular‐weight films contained smaller and more sparsely distributed crystallites embedded with a higher fraction of amorphous chains, which facilitated more uniform stress distribution, enhanced molecular reorientation, and markedly improved stretchability. Consistent with these structural characteristics, the extracted rS values increased monotonically with molecular weight, following the order P4 (11.5) > P3 (4.16) > P2 (2.78) > P1 (1.19). Under 100% strain, the mobilities of P3 and P4 decreased by approximately ∼30%, and 10%, respectively, and recovered to 85% and 100% upon strain release. In contrast, the mobilities of lower molecular polymers P1 and P2 decreased by ∼50% and further declined by an additional ∼10% after strain release [119]. Collectively, these results unambiguously establish higher molecular weight as a key determinant for achieving enhanced stretchability while preserving charge transport in polymer semiconductors.

### 2.3 Thiophene‐Flanked DPP for Biological Applications

Beyond the advantages discussed above in stretchable electronics, thiophene flanking groups also play a central role in enabling the intrinsic biocompatibility of DPP‐based systems, thereby facilitating their integration with biological environments and driving the emergence of bioelectronics [35, 120, 121]. In particular, by imparting chemical stability under biological conditions, spectral tunability, biocompatibility, and interfacial affinity, thiophene‐flanked DPP materials provide a versatile structural platform that can be readily tailored through side‐chain engineering or backbone functionalization to meet the stringent requirements of biological systems [122, 123]. Consequently, thiophene‐flanked DPP‐based OSCs have attracted considerable attention for bio‐related applications, where their implementation predominantly relies on direct molecular functionalization and nanoparticle formation.

**FIGURE 6:**
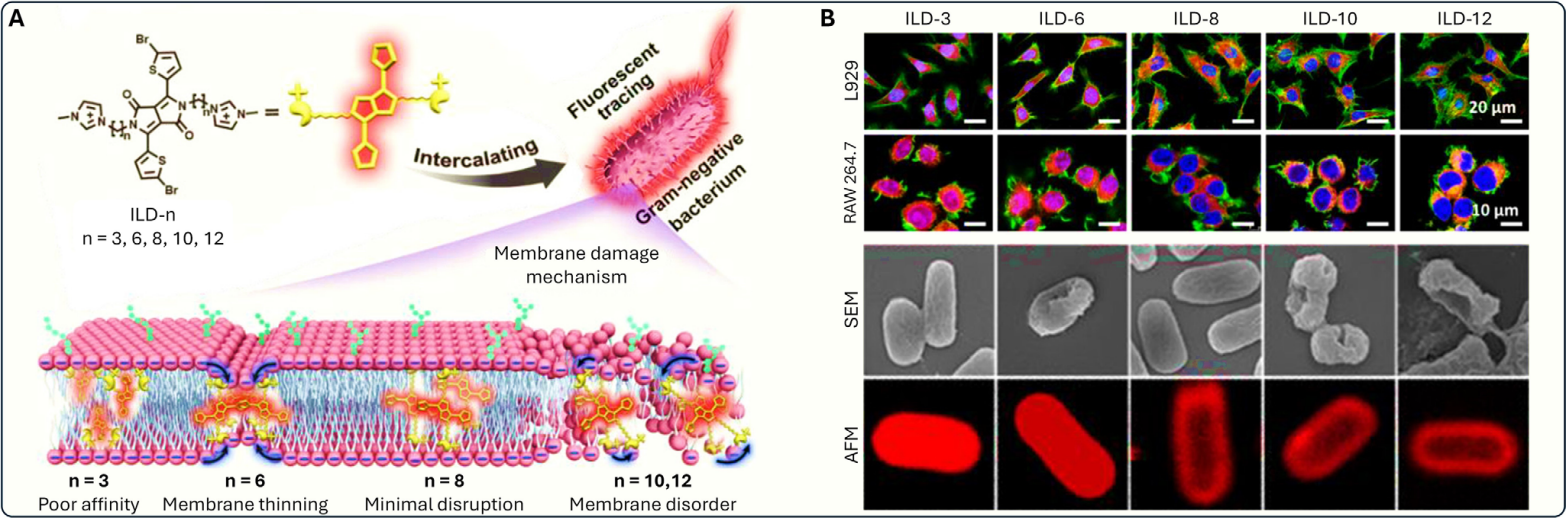
(A) Membrane damage mechanism of different ILDs against Gram‐negative bacteria. (B) Confocal laser scanning microscopy (CLSM) images of L929 and RAW 264.7 cells treated with ILDs. (Green: phalloidin‐FITC‐labeled cytoskeleton; blue: DAPI‐labeled nucleus; and red: ILDs). SEM and AFM images of *Escherichia coli* after treatment with ILDs. Adapted with permission [125]. Copyright 2020, ACS.

Within strategies based on direct structural modification, a primary objective is to enhance interactions with biological entities at the molecular and cellular levels. Du et al. introduced lysine‐functionalized side chains into DPP polymers to improve adhesion to human cells, thereby enabling direct neuronal cell growth on the material surface and highlighting the potential of DPP‐based OSCs for in vivo and cell‐integrated bioelectronic devices [124]. In another representative study, Zheng et al. incorporated antibacterial ionic liquid moieties into the side chains of DPP derivatives, referred to as ionic liquid‐functionalized DPPs (ILDs; Figure 6A). Their results revealed that the antibacterial activity against Gram‐negative bacteria is highly sensitive to molecular size, which is governed by the flexible spacer length between the DPP core and the ionic termini. Specifically, the smallest derivative, ILD‐3, exhibited weak membrane affinity and correspondingly poor antibacterial activity. In contrast, ILD‐6 and ILD‐8 were sufficiently large to span the lipid bilayer of cell membrane, however, ILD‐6 induced membrane thinning, whereas ILD‐8 caused minimal structural perturbation. For the largest derivatives, ILD‐10 and ILD‐12, dynamic fluctuations within the lipid bilayer resulted in pronounced disruption of membrane ordering. These distinct interaction mechanisms were consistently validated through in vitro, in silico, and in vivo studies, as evidenced by SEM and AFM analyses (Figure 6B). Antibacterial assays further demonstrated that both membrane thinning and membrane disruption contribute to antibacterial efficacy, with ILD‐6 (membrane thinning) and ILD‐12 (membrane disruption) exhibiting the highest activities. Notably, these membrane‐interaction mechanisms were not observed for Gram‐positive bacteria or eukaryotic cells. Confocal laser scanning microscopy (CLSM) images showed that all ILDs readily entered the cytoplasm of these cells without clear colocalization at the cell membrane (Figure 6B). These findings indicate that ILDs exhibit selective membrane interactions with Gram‐negative bacteria, providing valuable guidance for the rational design of next‐generation antibacterial agents [125].

To access a broader spectrum of biological processes, the electron‐deficient DPP core has also been incorporated into conventional fully donor‐derived conjugated oligoelectrolytes. By appending ionic groups to the termini of an otherwise hydrophobic DPP‐based conjugated core, the resulting molecule (COE‐KP) effectively mimics the spatial distribution of charged and hydrophobic domains characteristic of phospholipid bilayers in cell membrane, thereby achieving prolonged membrane retention. Importantly, the optical activity of DPP‐conjugated units imparts favorable photophysical properties for bioimaging functions. Moreover, the DPP core is sensitive to hydrogen‐bonding interactions, allowing COE‐KP to report variations in lipid composition within vesicle membranes through interactions with surrounding water solute. As a result, this class of materials exhibit broad applicability in bioimaging, including long‐term studies of cell growth and proliferation, visualization of subcellular organelles, and investigations of lipid bilayer‐based vesicles, thereby significantly expanding the utility of conjugated oligoelectrolytes in biological systems [126].

For bioelectronic applications, semiconducting materials are generally required to exhibit both efficient charge transport and strong interactions with biological systems. However, structural modifications introduced to enhance bio‐interfacial interactions often disrupt molecular packing, thereby compromising charge transport properties. To address this trade‐off, Li et al. developed a versatile “click‐to‐polymer” synthesis strategy that enables the postfunctionalization of presynthesized polymer backbones with diverse functional groups. This approach largely preserves the intrinsic backbone packing and charge carrier transport, while simultaneously introducing new functionalities tailored for human‐integrated electronics. The utility of this strategy was demonstrated through the realization of photopatternable polymers and biochemical sensing platforms, highlighting its potential for advanced bioelectronic applications [33].

**FIGURE 7:**
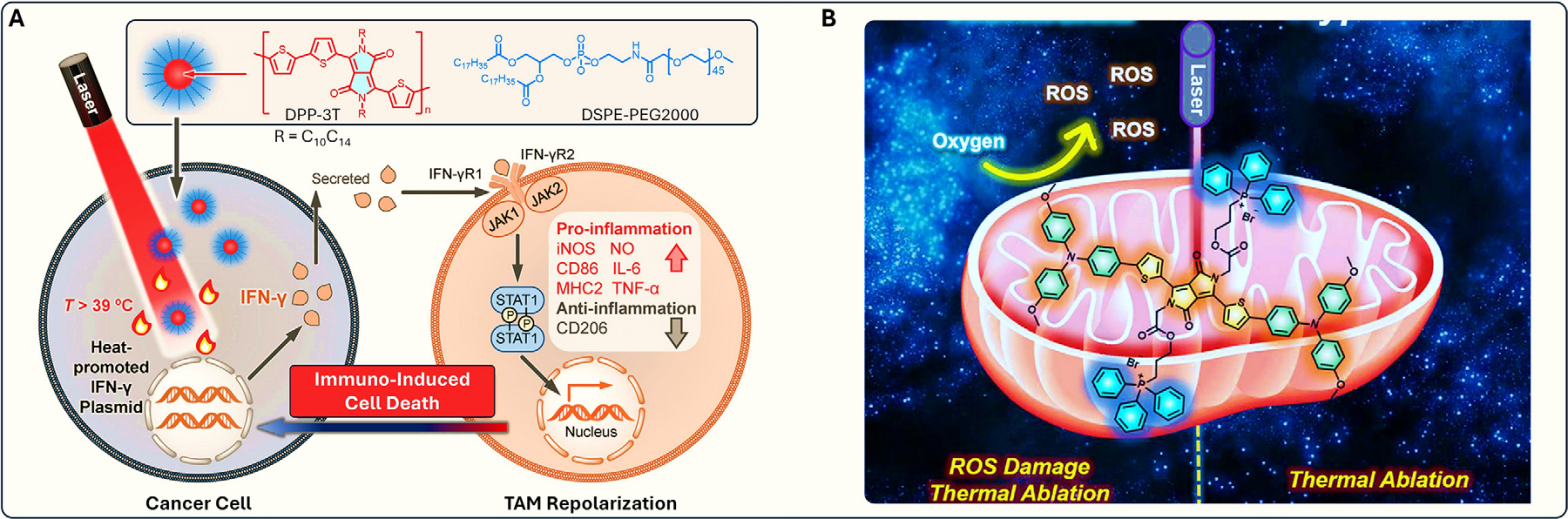
(A) Illustration of the remote‐control cancer immunotherapy mediated by photothermal conjugated polymer nanoparticles. Adapted with permission [128]. Copyright 2021, Wiley. (B) Graphical illustration of a mitochondria‐targeted nanotheranostic agent. Adapted with permission [129]. Copyright 2021, Elsevier.

Another important strategy for deploying DPP‐based materials in biological systems involves their formulation into nanoparticles, which enables remote and spatiotemporally controlled therapeutic activation while minimizing off‐target effects prior to cellular internalization, an especially desirable feature for cancer immunotherapy [122, 127]. Within this framework, DPP‐based nanomaterials have emerged as versatile platforms for multimodal disease imaging, diagnosis, and therapy. For example, Fu et al. developed a near‐infrared (NIR) light‐activated optogenetic system by integrating photothermally responsive DPP nanoparticles (DPP‐NPs) with a heat‐inducible interferon‐γ (IFN‐γ) plasmid (Figure 7A) [128]. Upon NIR irradiation, the DPP‐NPs generate localized heat that activates IFN‐γ transcription via the HSP70 promoter. The secreted IFN‐γ subsequently repolarizes tumor‐associated macrophages, thereby reshaping the immunosuppressive tumor microenvironment and promoting cancer cell elimination. These activated macrophages further enhance phagocytosis and adaptive immune responses, demonstrating that this system enables noninvasive and precisely controlled activation of antitumor immunity, and highlighting its promise for cancer immunotherapy. Despite the advantages of phototherapy, maximizing photon utilization to achieve complete tumor suppression while preventing recurrence remains challenging. To address this issue, Shin et al. developed a mitochondria‐targeted phototheranostic nanoformulation based on DPP materials (Figure 7B) [129]. From a molecular design perspective, incorporation of a triphenylphosphonium (TPP) moiety into the DPP side chains facilitates both singlet‐to‐triplet intersystem crossing and nonradiative decay from the excited state, thereby enabling the simultaneous activation of photodynamic therapy (PDT) and photothermal therapy (PTT). This dual phototherapeutic mechanism maximizes photon utilization for efficient tumor ablation. Under single‐wavelength (690 nm) laser irradiation, the resulting nanoparticles enable dual‐mode photoacoustic and NIR fluorescence imaging, along with switchable PDT/PTT by balancing radiative, energy‐transfer, and nonradiative pathways. Compared with nontargeted controls, these nanoparticles exhibit enhanced tumor accumulation and mitochondrial localization, enabling effective PDT under normoxic conditions and PTT under hypoxic conditions. Consequently, this strategy achieves potent tumor inhibition without observable side effects and provides an effective solution to overcome hypoxia‐associated resistance in PDT. Beyond the phototherapy‐based approach, Xu et al. reported a supramolecular “ice cell and fire nanomedicine” platform that synergistically integrates PTT, gas therapy, and immunotherapy to suppress cancer recurrence and metastasis [130]. In this system, a J‐aggregated DPP photothermal agent enabled efficient tumor ablation under safe laser power densities, while a co‐loaded nitric oxide donor released cytotoxic gas to degrade the tumor extracellular matrix and enhance immune cell infiltration. Furthermore, anchoring the nanomedicine to frozen cancer cells significantly amplified antitumor immune responses. As a result, this multifunctional platform effectively suppressed primary tumors, distant tumors, and lung metastases following a single treatment, demonstrating its potential for comprehensive and durable cancer therapy.

To further improve the precision of phototheranostic systems, expanding probe responsiveness to multiple pathological factors and excitation wavelengths is highly desirable. In this regard, Yang et al. developed a dual‐stimuli semiconducting polymer nanoprobe incorporating DPP units for activatable NIR‐II photoacoustic imaging and PTT [131]. The nanoprobe selectively responds to the co‐presence of nitric oxide and acidic conditions, triggering a molecular transformation that enhances NIR‐II absorption and photoacoustic signal output. Such dual‐factor activation enables highly specific imaging and efficient PTT with improved signal‐to‐noise ratios, deep tissue penetration, and minimal side effects. More broadly, this work provides a generalizable molecular design framework for constructing biomarker‐activatable nanoprobes, advancing the development of precise deep‐tissue phototheranostic platforms. Beyond oncology, atherosclerosis therapy presents additional challenges, as early‐stage lesions are often difficult to diagnose due to the absence of obvious clinical symptoms. To address this unmet need, Ma et al. developed a lesion‐targeting theranostic nanoplatform based on DPP materials, termed PLCDP@PMH, for the noninvasive diagnosis and treatment of atherosclerosis [132]. This nanoparticle system enables plaque‐specific photoacoustic imaging combined with reactive oxygen species (ROS)/matrix metalloproteinase (MMP) responsive release of a multifunctional therapeutic complex for integrated lipid management. In particular, the strong intrinsic photoacoustic contrast of the DPP component allows precise identification and diagnosis of atherosclerotic plaques. In vivo studies demonstrated that this strategy effectively suppresses inflammation, reduces lipid uptake, enhances cholesterol efflux, and promotes lipid removal, leading to accurate plaque visualization, inhibition of lesion progression, and even regression of established plaques under a non‐high‐fat diet. These results underscore the strong potential of DPP‐based nanoplatforms for early‐stage atherosclerosis theranostics.

**FIGURE 8:**
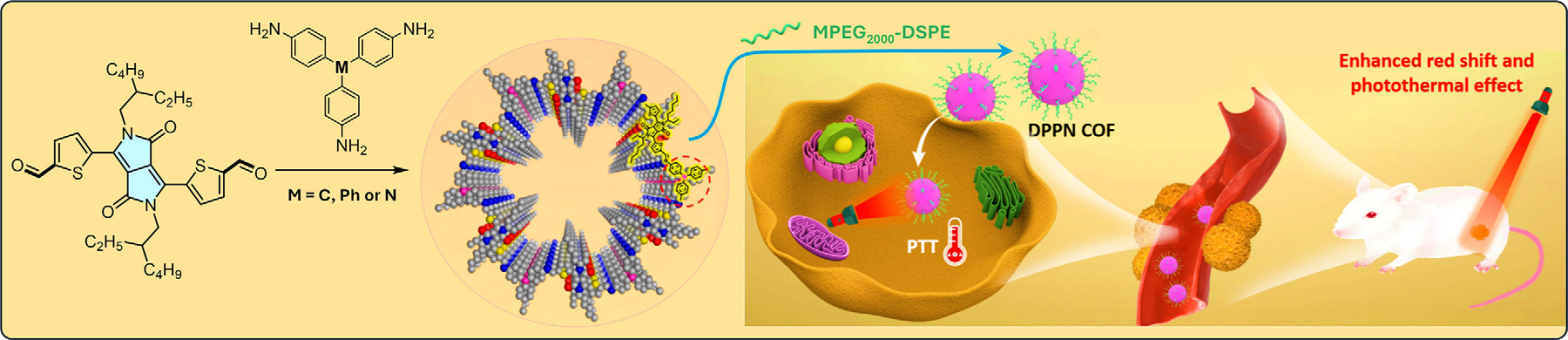
DPPT‐based nanoscale covalent organic frameworks (COFs) for photothermal ablation of tumors. Adapted with permission [133]. Copyright 2021, ACS.

Beyond molecular and polymeric systems, covalent organic frameworks (COFs) have also attracted considerable interest in biomedical applications owing to their large surface areas and intrinsic porosity, which enable efficient loading of hydrophobic drugs and facilitate ROS diffusion during PDT. However, the poor solubility of COFs often compromises their colloidal stability in biological media, as conventional sonication‐based processing typically produces exfoliated nanosheets with partially collapsed or disrupted pore structures. To overcome this limitation, Xia et al. developed a general synthetic strategy for preparation of monodisperse, size‐controllable COF nanoparticles with uniform spherical morphology and robust colloidal stability (Figure 8) [133]. This approach is based on tuning the catalyst concentration and the electron‐donating properties of the COF precursors, allowing precise control over particle size and optical properties. As a result, the absorption spectra of these COF nanoparticles can be engineered to extend into the NIR‐II biowindow, enabling them with efficient photothermal activity and effective tumor growth inhibition under laser irradiation. This strategy offers a versatile platform for integrating diverse molecular systems into COF nanostructures for PTT, while holding broader potential for biomedical and catalytic applications.

### 2.4 Thiophene‐Flanked DPP in Perovskite Solar Cells

Perovskite solar cells have emerged as one of the most promising photovoltaic technologies in recent years [50]. However, their development is hindered by the limited availability of efficient electron transport materials (ETMs), as current device architectures still predominantly rely on fullerene derivatives. This reliance not only increases manufacturing costs but also constrains large‐scale production and the realization of flexible devices. In this context, NFAs have attracted increasing attention as viable alternatives. A key challenge, however, lies in identifying suitable NFAs that can simultaneously provide efficient vertical electron transport and effective interfacial passivation at the perovskite/ETM interface [134, 135]. Thiophene‐flanked DPP materials offer several distinctive advantages arising directly from the role of the flanking units. On one hand, their extensive study has enabled the realization of exceptionally high charge‐carrier mobilities [79]. More importantly, the thiophene flanking groups allow precise modulation of molecular packing through rational design, which is more conducive to efficient vertical charge extraction and transport than the isotropic packing of fullerene‐based materials. Furthermore, both the thiophene flanking units themselves and the diverse heteroatoms incorporated within thiophene‐flanked DPP frameworks facilitate effective passivation of perovskite surfaces, thereby enhancing the operational stability of perovskite photovoltaic devices.

**FIGURE 9:**
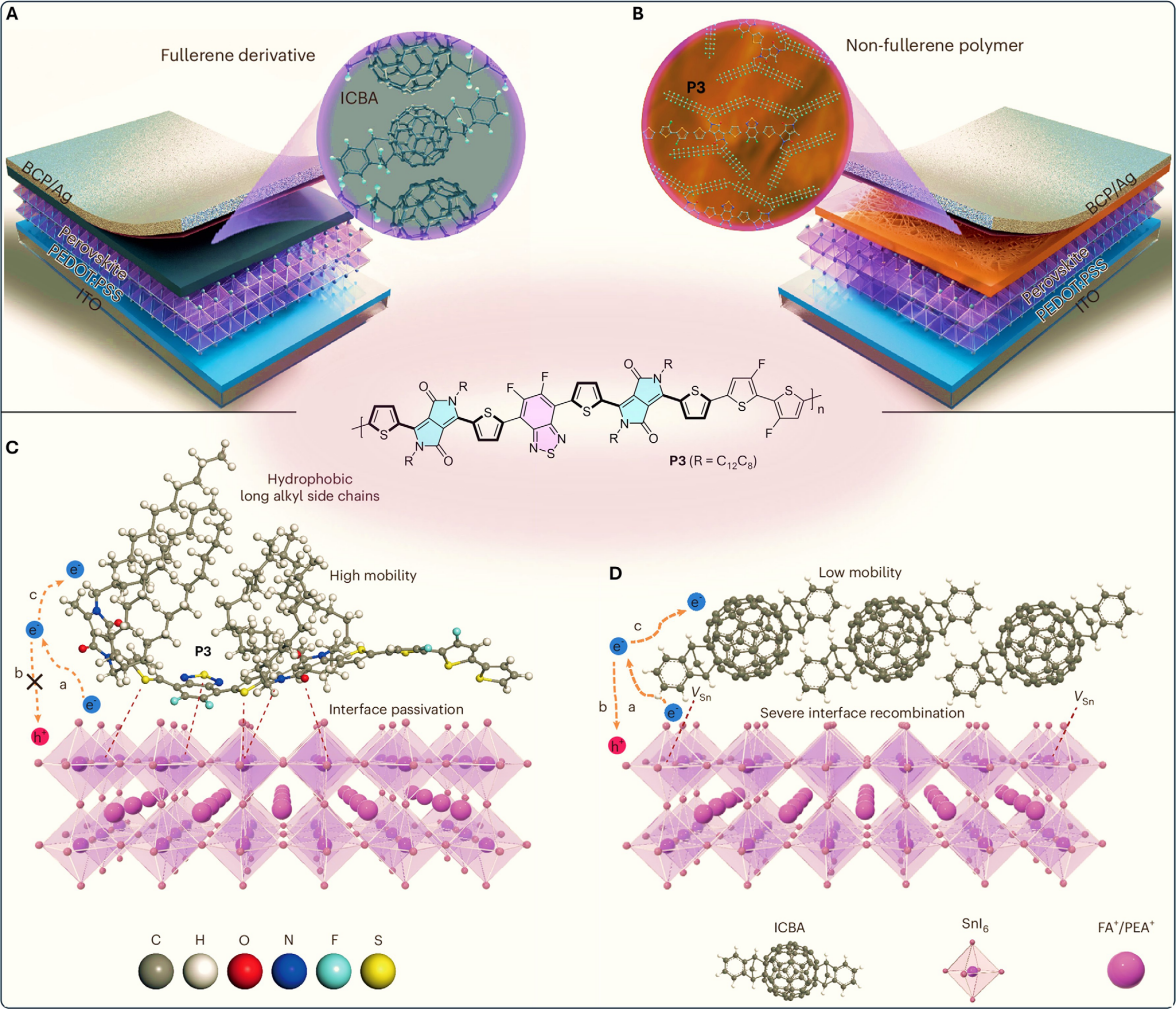
Representative device architectures of tin‐based perovskite solar cells employing. (A) fullerene and (B) nonfullerene electron transport layers. Schematic illustrations of interfacial interactions between tin‐based perovskites and (C) the fullerene‐based acceptor ICBA or (D) the nonfullerene acceptor P3. Adapted with permission [136]. Copyright 2025, Nature.

Compared with lead‐based counterparts, tin‐based perovskites offer superior environmental compatibility, making the development of high‐performance, large‐area tin‐based perovskite solar cells particularly important for sustainable photovoltaics. To achieve this goal, nonfullerene ETMs are especially attractive, as they can form more continuous and conformal interfaces with the perovskite layer than conventional fullerene acceptors. Li et al. proposed a triple‐acceptor design strategy based on DPPT units to develop high‐performance n‐type semiconductors. Using this strategy, a new polymer (P3, structure shown in Figure 9) was synthesized, exhibiting a high electron mobility of 1.29 cm^2^ V^−1^ s^−1^, low material cost, and excellent structural tunability, features that collectively surpass those of the widely used fullerene derivative ICBA. When applied as the ETM in tin‐based perovskite solar cells (Figure 9A,B) to replace ICBA, P3 enabled a record power conversion efficiency of 16.06%, significantly outperforming devices based on fullerene ETMs (11.74%) [136]. The enhanced device performance is primarily attributed to the strong interfacial interactions between the tin perovskite and the nonfullerene ETM, arising from the abundant C−S, C−N, and C−F bonds present in the polymer backbone. In particular, nonfullerene ETMs interact more effectively with Sn^2+^ ions in the perovskite layer, thereby suppressing the oxidation of Sn^2+^ and inhibiting iodide migration. As a result, electron extraction from the perovskite to the ETM (process *a* in Figure 9C) is promoted, while undesired hole back‐transfer from the ETM to the perovskite (process *b*) is effectively suppressed (Figure 9C). In contrast, fullerene‐based ETMs exhibit weaker interfacial interactions, leading to a slower electron extraction and a more pronounced hole back‐transfer (Figure 9D). Moreover, charge transport within the ETM layer itself (process *c*) proceeds substantially faster in nonfullerene ETMs, benefiting from electron mobilities that are approximately three orders of magnitude higher than those of fullerene counterparts. Notably, the intrinsic hydrophobicity of nonfullerene acceptors, when used as electron transport layers, further contributes to enhanced operational stability in fullerene‐free perovskite solar cells. These combined advantages highlight the significant potential of nonfullerene ETMs in advancing stable, efficient, and industrially scalable perovskite photovoltaic technologies.

Overall, advances in thiophene‐flanked DPP system highlight how a well‐established flanking unit can function as a “platform chemistry,” wherein its intrinsic electronic and structural characteristics enable diverse secondary modifications and ultimately support a broad range of emerging applications.

### 2.5 Furan‐ and Selenophene‐Flanked DPP Systems

Beyond thiophene as the conventional flanking unit for DPP, alternative heterocycles such as furan and selenophene have been explored to modulate charge carrier polarity in organic electronic materials, owing to their different electron‐donating strengths. Furan functions as a relatively weak electron donor, whereas selenophene is a stronger donor. Notably, the longer C‐Se bond length in selenophene enhances quinoid character along the conjugated backbone, giving rise to unconventional and diversely tunable charge transport characteristics. Liu et al. demonstrated that tailoring the electron affinity of DPP flanking groups provides a simple yet effective means of regulating charge carrier polarity. In p‐dominant ambipolar polymers bearing furan or selenophene flanking unit, the furan‐based polymer exhibited a markedly lower hole‐to‐electron mobility ratio (*µ*_h_/*µ*_e_ = 1.9) compared to its selenophene‐based counterpart (*µ*_h_/*µ*_e_ = 26.7) [137]. This pronounced disparity is consistent with the weaker donor character of furan and the stronger donor nature of selenophene. However, such charge carrier polarity is largely intrinsic to the polymer backbone, making precise and continuous tuning challenging. To overcome this limitation, the authors introduced highly soluble ionic additives, enabling fine control over doping concentration throughout the polymer/additive volume. Systematic investigations of doping levels in furan‐ and selenophene‐based n‐dominant polymers revealed that transport behavior can be precisely modulated over a broad range, spanning p‐dominant, balanced ambipolar, n‐dominant, and ultimately unipolar n‐type characteristics [138]. Comparative analyses across both the p‐dominant and n‐dominant systems further indicate that selenophene enhances hole transport in p‐dominant regimes due to its stronger electron‐donating ability, whereas in n‐dominant systems it more effectively exerts quinoid effects, thereby promoting electron transport. This dual functionality is particularly important for the development of complementary circuits, as it enables versatile and precise control over charge carrier polarity within a single materials platform.

### 2.6 Thiazole‐Flanked DPP Systems

**FIGURE 10:**
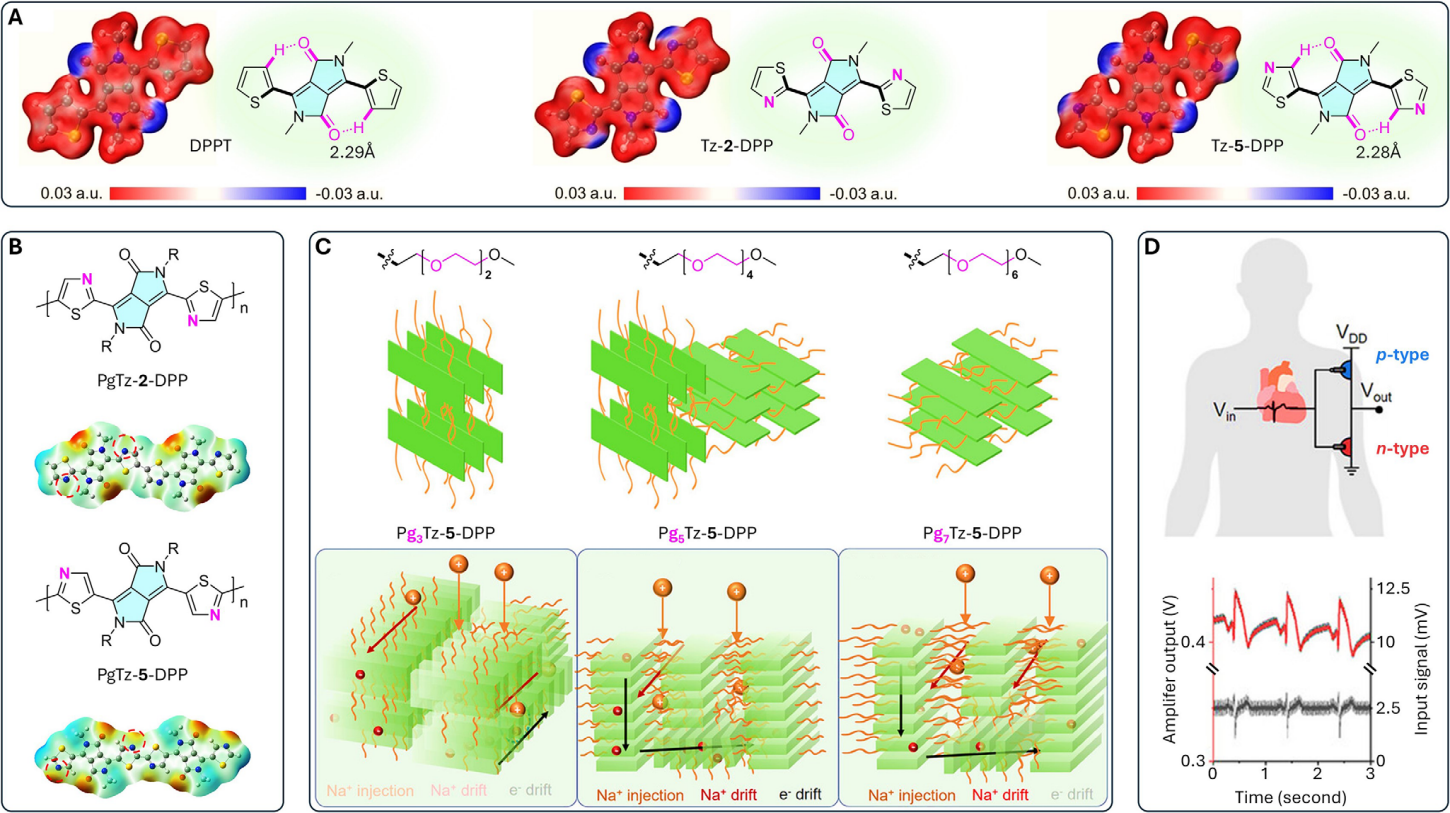
(A) Electrostatic potential (ESP) surfaces of methyl‐substituted DPPT, Tz‐2‐DPP, and Tz‐5‐DPP. Adapted with permission [145]. Copyright 2023, Wiley. (B) Molecular structures of PgTz‐2‐DPP and PgTz‐5‐DPP, together with the ESP maps of corresponding methyl‐substituted dimers. (C) Side‐chain‐induced backbone orientations in the PgTz‐5‐DPP series, and a schematic illustration of the interplay between side‐chain engineering and device operation. (D) Demonstration of electrocardiogram (ECG) signal recording using an organic inverter as the amplifier. Adapted with permission [148]. Copyright 2025, Wiley.

In addition to furan and selenophene, thiazole has emerged as another promising flanking unit for DPP‐based materials [137, 138, 139, 140, 141, 142, 143, 144, 145, 146, 147, 148]. As illustrated in Figure 10A, in comparison with thiophene‐flanked DPP (DPPT), the incorporation of a nitrogen atom containing thiazole in Tz‐2‐DPP suppresses hydrogen‐bonding interactions between the flanking unit and the DPP core. Moreover, electrostatic repulsion between the negatively charged nitrogen atom and the carbonyl oxygen of the DPP unit favors a *trans* backbone conformation, in contrast to the conformation typically adopted by DPPT. By relocating the nitrogen atom to the 5‐position to form Tz‐5‐DPP, hydrogen bonding interactions are restored, stabilizing the *cis* conformation instead [145]. These distinct conformational preferences lead to substantial differences in backbone planarity, intermolecular interactions, and electronic coupling, ultimately leading to substantially different device performances across a range of organic electronic devices.

Ma et al. investigated how these conformational differences influence organic electrochemical transistor (OECT) performance by synthesizing two thiazole‐flanked DPP homopolymers, PgTz‐2‐DPP and PgTz‐5‐DPP (Figure 10B) [148]. Their study revealed that the regiospecific position of the sp^2^‐hybridized nitrogen atom in the repeating thiazole unit plays a decisive role in governing polymer solvation behavior and molecular packing. This effect becomes particularly pronounced in the polar solvent hexafluoroisopropanol, where the PgTz‐5‐DPP series exhibits enhanced solvation. This improved solvation facilitates structural reorganization during solvent evaporation, thereby promoting stronger interchain interactions compared to the PgTz‐2‐DPP series, which remain aggregated in solution. As a result, PgTz‐5‐DPP displays improved conjugation efficiency, delivering a reduced π–π stacking distance (3.49 Å) than that of PgTz‐2‐DPP (3.53 Å). Furthermore, the introduction of glycol side chains onto the PgTz‐5‐DPP backbone revealed that side‐chain length strongly influences molecular packing during film formation. Shorter glycol chains favor an edge‐on orientation, whereas longer glycol chains promote a transition toward a face‐on orientation. This tunability in molecular orientation is particularly advantageous for OECT operation, which requires both efficient vertical ion injection (favored by face‐on orientation) and lateral ion drift and electronic transport (favored by edge‐on orientation), as illustrated in Figure 10C. Consequently, Pg_5_Tz‐5‐DPP adopts a bimodal microstructure comprising both face‐on and edge‐on domains, enabling an optimized balance between ionic and electronic transport. This structural synergy results in exceptional n‐type device performance, including a geometrically normalized transconductance of 31.9 S cm^−1^, a figure of merit (µC*) of 96.3 F cm^−1^ V^−1^ s^−1^, and a low threshold voltage of 0.31 V, placing this material among the highest performing n‐type organic mixed ionic‐electronic conductors (OMIECs). Building on these advances, an organic complementary inverter fabricated from the optimized n‐type Pg_5_Tz‐5‐DPP and a reported p‐type polymer exhibited a voltage gain of 198 V V^−1^, enabling effective amplification of electrocardiogram (ECG) signals and significantly improving signal quality (Figure 10D). Overall, this work establishes clear structure‐property guidelines for the rational design of high‐performance thiazole‐flanked DPP‐based n‐type OMIECs for bioelectronic applications.

**FIGURE 11:**
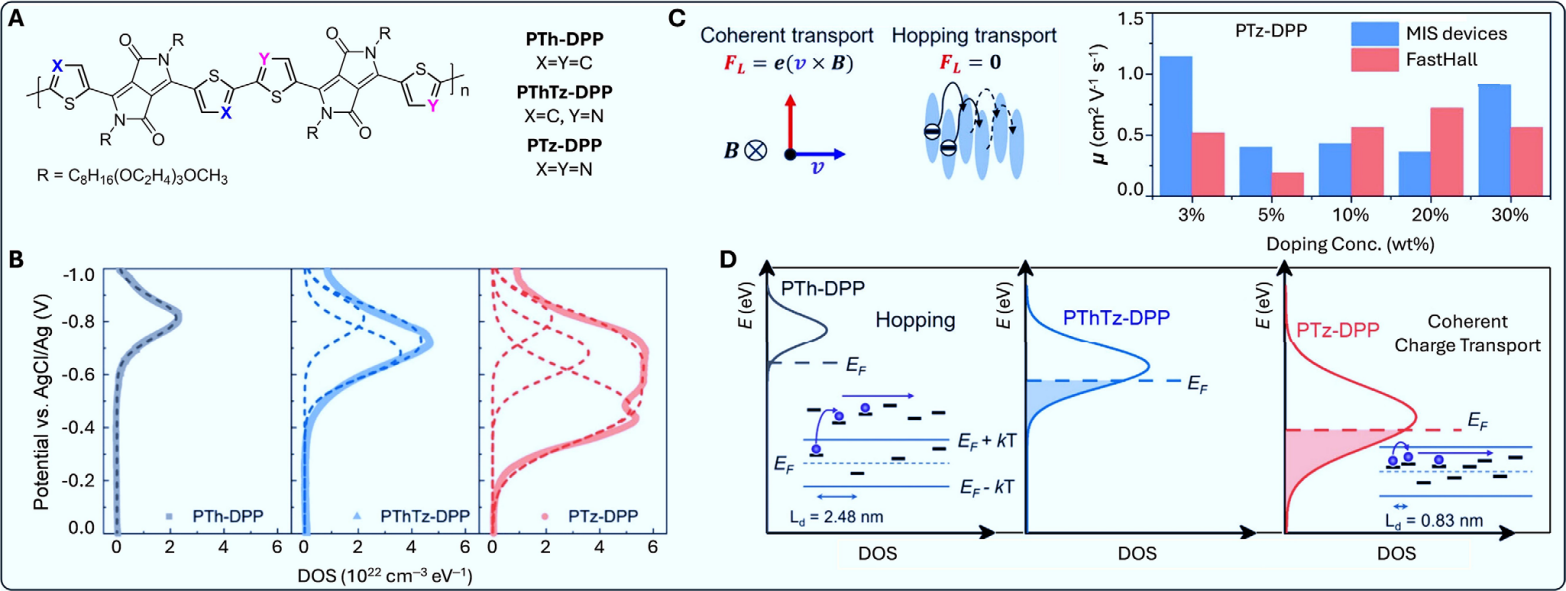
(A) Molecular structures of PTh‐DPP, PThTz‐DPP, and PTz‐DPP. (B) Density‐of‐states (DOS) distributions of undoped polymer films. (C) Charge carrier mobilities extracted from Mott–Schottky analysis and FastHall measurements of PTz‐DPP films. (D) DOS distributions of doped polymer films. Adapted with permission [147]. Copyright 2025, ACS.

Building on the advantageous characteristics of the Tz‐5‐DPP motif, particularly its high solvation capability and variable molecular orientation, both of which are beneficial for doping‐related investigations, the authors systematically examined the influence of the 5‐position thiazole loading ratio on the thermoelectric performance of DPP‐based materials. To this end, three polymers, PTh‐DPP, PThTz‐DPP, and PTz‐DPP, were developed (Figure 11A) [147]. These polymers were prepared via direct arylation polymerization (DAP), a particularly attractive synthetic route for n‐type organic semiconductors because it bypasses the longstanding challenges associated with electron‐deficient monomers that require bistannyl or diboronic functionalization.

Although DAP often suffers from low C–H bond reactivity and poor selectivity in C–H activation, the incorporation of thiazole units effectively mitigates these issues. In particular, the imine nitrogen atom at the 5‐position of thiazole enhances C–H bond reactivity while simultaneously suppressing undesired coupling at the nitrogen site, an adverse reaction pathway that would otherwise be encountered if this position were occupied by carbon. As a result, thiazole‐flanked DPP monomers exhibit both high reactivity and excellent selectivity in C–H activation, enabling the efficient synthesis of a range of polymers with molecular weights spanning 10–100 kDa [145].

As the polymer backbone evolves from PTh‐DPP to PThTz‐DPP and ultimately to PTz‐DPP, the progressive increase in nitrogen content exerts a pronounced influence on the density of states (DOS) (Figure 11B) [147]. PTh‐DPP exhibits a single DOS peak. Upon partial substitution of thiophene with thiazole in PThTz‐DPP, an additional energy band emerges, indicating increased energetic heterogeneity. Full substitution with thiazole units in PTz‐DPP further broadens and delocalizes the DOS, resulting in three distinct peaks. This evolution indicates the formation of deeper energy centres and a higher overall site density in PTz‐DPP, both of which are conducive to more efficient doping and the generation of free charge carriers with enhanced mobility.

FastHall measurements further elucidate the charge transport mechanism in the doped state. Doped PTh‐DPP films exhibited Hall‐effect signals within the noise level, indicative of a hopping‐dominated transport regime. In contrast, doped PTz‐DPP films displayed pronounced Hall‐effect signals on the order of several microvolts, corresponding to charge carrier mobilities ranging from 0.2 to 0.7 cm^2^ V^−1^ s^−1^, values comparable to those obtained from metal‐insulator‐semiconductor (MIS) device measurements (Figure 11C). These results suggest that a substantial fraction of charge carriers in doped PTz‐DPP undergo coherent transport that is further corroborated by analysis of the Fermi level (E_F_) positions (Figure 11D). Specifically, in doped PTh‐DPP, the average site spacing near E_F_ is significantly larger than the polymer repeating unit, necessitating thermally activated hopping to access neighboring states at higher energies. In contrast, PTz‐DPP exhibits a much shorter site spacing near E_F_, comparable to the repeating unit length, thereby enabling coherent charge transport in the vicinity of the Fermi level.

Collectively, these results clearly demonstrate the promise of thiazole‐based DPP systems for the development of high‐performance n‐type organic semiconductors. In particular, their compatibility with direct arylation polymerization offers a powerful platform for incorporating a wide range of brominated electron‐deficient acceptors, thereby enabling fine‐tuning of the electron affinity and electronic structure of the resulting polymer backbones.

## 3 Six‐Membered Aromatic Flanking of DPP

Six‐membered aromatic flanking units attached to the DPP core generally introduce greater steric hindrance than their five‐membered counterparts, resulting in increased backbone torsion. While such steric effects are often regarded as detrimental to π‐conjugation, they can be judiciously exploited for specific applications. A representative example is phenyl‐flanked DPP, whose intrinsic structural twist is advantageous for geometric modulation, fluorescence tuning, and porosity control in COFs.

### 3.1 Phenyl‐Flanked DPP Systems

Regeni et al. leveraged the conformation‐induced stacking motifs of phenyl‐flanked DPP, in combination with N‐based donor terminals capable of coordinating Pd ions, to construct a series of metallosupramolecular self‐assemblies [149]. They demonstrated that the interplay among donor terminal selection, backbone steric torsion, and nitrogen coordination position governs the formation of discrete supramolecular architectures. These distinct assemblies enabled fine modulation of photoluminescence quantum yield (PLQY), with a heteroleptic, doubly bridged Pd_2_L_2_L’_2_ complex exhibiting a notably high PLQY of 51%. In organic electronic systems, molecular assembly critically influences charge transport across multiple length scales, from interfacial regions to bulk active layers. Consequently, well‐defined strategies for tuning supramolecular organization are expected to advance the development of diverse electronic devices. Moreover, in emerging doping‐related technologies, such as organic thermoelectrics and OECTs, the resulting three‐dimensional architectures are particularly attractive, as they can simultaneously provide sufficient free volume for efficient dopant incorporation while preserving continuous charge‐transport pathways for both electronic and ionic transport.

Kim et al. reported another supramolecular system based on phenyl‐flanked DPP, employing a host–guest strategy for biolabeling and advanced optical microscopy applications [37]. By introducing positively charged terminal groups to impart water solubility and incorporating linkers into the side chains, the resulting DPP derivatives enabled fluorescence detection of bioconjugates involving antibodies and nanobodies. Upon complexation with the host protein cucurbit[7]uril (CB7), the DPP fluorophores exhibited significantly enhanced photostability and an approximately twelvefold increase in emission intensity. This marked improvement translated into substantially higher brightness and image quality in two‐color confocal as well as stimulated emission depletion (STED) microscopy. Beyond optical performance, CB7 functionalization also facilitated cellular permeation of the fluorescent DPP probes, enabling effective labelling of fixed cells. These results clearly illustrate that rational structural modification and supramolecular engineering of phenyl‐flanked DPPs can unlock new functionalities, encouraging their adoption across multiple disciplines.

The steric hindrance associated with phenyl‐flanked DPPs has also been shown to suppress excessive molecular aggregation on TiO_2_ surfaces in dye‐sensitized solar cells, thereby mitigating charge recombination between injected electrons and the electrolyte. Building on this feature, Romito et al. demonstrated that terminal functionalization of phenyl‐flanked DPPs with TEMPO radicals can eliminate the need for sacrificial electron donors in dye‐sensitized photocatalysis [150]. Conventional dye‐sensitized photocatalytic systems typically rely on sacrificial donors to regenerate oxidized dyes, which are often toxic, costly, and efficiency‐limiting. By integrating an intrinsic TEMPO photosensitizer, the resulting system enabled dual functionality of hydrogen evolution occurred at the solid–liquid interface, while alcohol oxidation to aldehydes proceeded in solution, with facile product separation. This work highlights the capacity of phenyl‐flanked DPPs to integrate multiple functional roles within a single molecular framework, pointing to promising opportunities for expanding DPP‐based materials into new application domains.

In a separate study focused on singlet fission, Kim et al. examined a phenyl‐flanked DPP core covalently linked to two pentacene units [151]. Unlike conventional static dimer systems, the incorporation of the phenyl‐flanked DPP imparted pronounced conformational flexibility due to the free rotation of the terminal pentacene moieties. This dynamic behavior gave rise to multiple rotational conformers, which in turn enabled diverse singlet‐fission pathways. Such confirmational adaptability underscores the potential of phenyl‐flanked DPPs as versatile molecular scaffolds, where steric design and conformational freedom can be harnessed to access a broad range of photophysical properties relevant to next‐generation optoelectronic and photonic applications.

Another notable conformational feature of phenyl‐flanked DPPs is their ability to undergo reversible aromatic‐quinoid transitions, endowing them with pronounced redox activity. In this context, Searle et al. demonstrated that such redox‐active behavior can enhance the performance of lithium‐sulfur (Li‐S) batteries [152]. Although Li‐S batteries offer exceptionally high theoretical gravimetric energy densities, their practical deployment is hindered by sluggish polysulfide redox kinetics. The incorporation of electron‐deficient phenyl‐flanked DPPs addressed this limitation by acting as redox mediators that facilitate electron transfer between polysulfide intermediates and the current collector. Specifically, the strong redox activity of the DPP molecules enabled rapid electron shuttling, thereby accelerating polysulfide conversion reactions, an effect unattainable with redox‐inactive additives. As a result, batteries incorporating these DPP‐based mediators exhibit markedly enhanced conversion kinetics and substantially increased discharge capacity, underscoring the potential of phenyl‐flanked DPPs as functional redox mediators in advanced energy‐storage systems.

**FIGURE 12:**
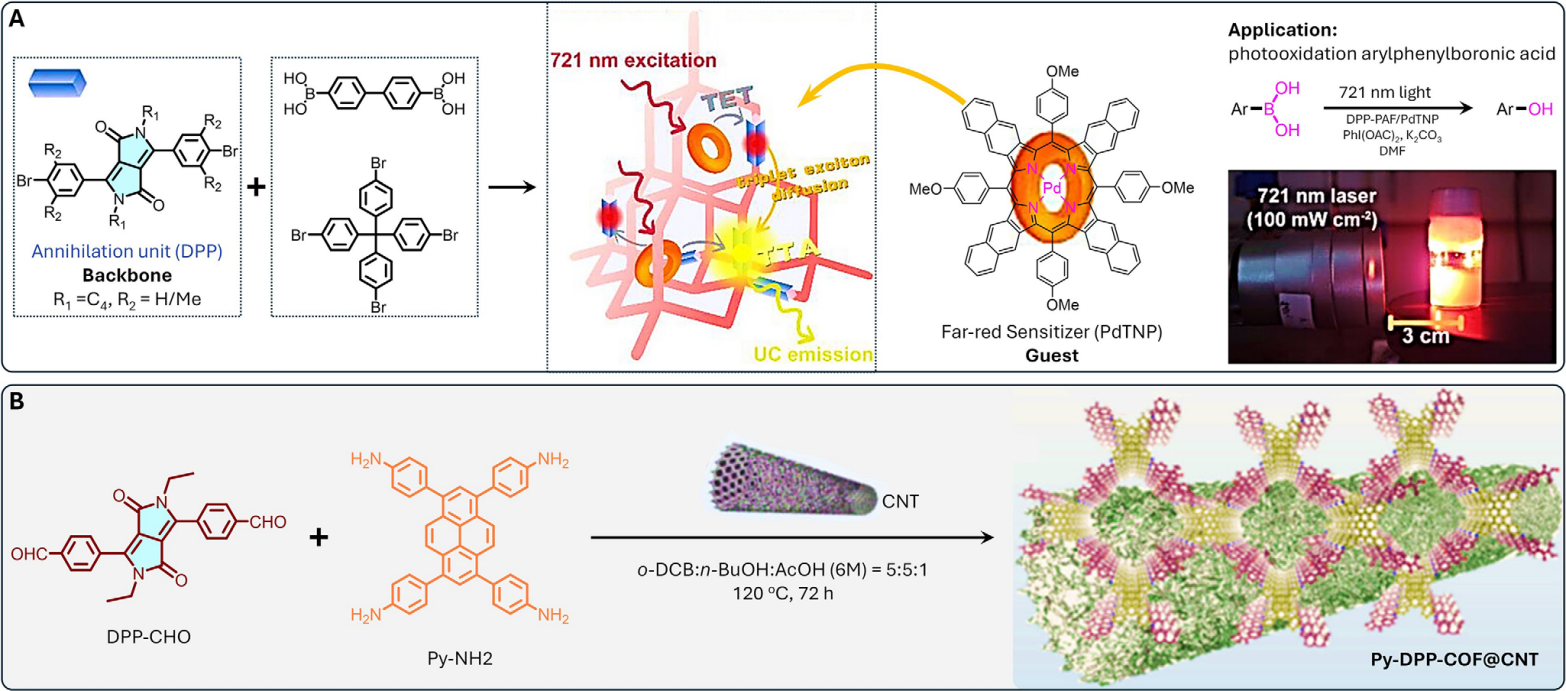
(A) Schematic illustration of excitable triplet‐triplet annihilation upconversion porous aromatic frameworks and their application in the photooxidation of arylphenylboronic acids to arylphenols. Adapted with permission [153]. Copyright 2025, Wiley. (B) Schematic illustration of the dynamic synthesis of COF@CNT. Adapted with permission [154]. Copyright 2021, ACS.

Beyond molecular systems, phenyl‐flanked DPPs have also been incorporated into COFs to introduce and amplify multifunctionality. Zhang et al. reported that, in porous aromatic frameworks loaded with a triplet photosensitizer (PdTNP), the steric hindrance of phenyl‐flanked DPPs, employed as triplet‐triplet annihilation units (Figure 12A), effectively disrupts extended π‐conjugation between adjacent DPP moieties and the framework [153]. This structural decoupling produces more homogeneous triplet exciton energy landscape, thereby facilitating long‐range exciton diffusion. As a result, the upconversion emission efficiency was enhanced by approximately 50‐fold, enabling markedly improved energy transfer efficiency in photocatalytic transformations, such as the conversion of boron compounds to alcohols. In another example, although the redox activity of isolated DPP molecules has been shown to enhance the discharge capacity of Li‐S batteries, as discussed above, the weak interlayer interactions and intrinsically low conductivity of DPP‐incorporated COFs can limit their practical electrochemical performance. To address these challenges, Xu et al. developed an in situ strategy to grow DPP‐COFs directly on the carbon nanotube (CNT) surfaces as shown in Figure 12B [154]. In this hybrid architecture, DPP units retain their role as redox mediators that promote polysulfide conversion, while CNTs provide strong interfacial interactions and highly conductive pathway, thereby enhancing overall electrocatalytic activity. Consequently, Li‐S batteries based on CNT‐modified DPP‐COFs outperformed those employing either pristine CNTs or pristine DPP‐COFs alone. Notably, at an optimized DPP‐COF@CNT content of 66 wt%, the system exhibited maximal electrocatalytic performance, delivering an ultralow capacity decay rate of only 0.042% over 1000 cycles and a high areal capacity of 8.7 mAh cm^−2^ at a sulfur loading of 10 mg cm^−2^ under lean electrolyte conditions (*E*/*S* = 5). This hybrid‐material strategy effectively broadens the applicability of DPP‐based COFs by synergistically integrating complementary components to mitigate the intrinsic limitations of individual components.

### 3.2 Pyridine‐ and Pyrazine‐Flanked DPP Systems

**FIGURE 13:**
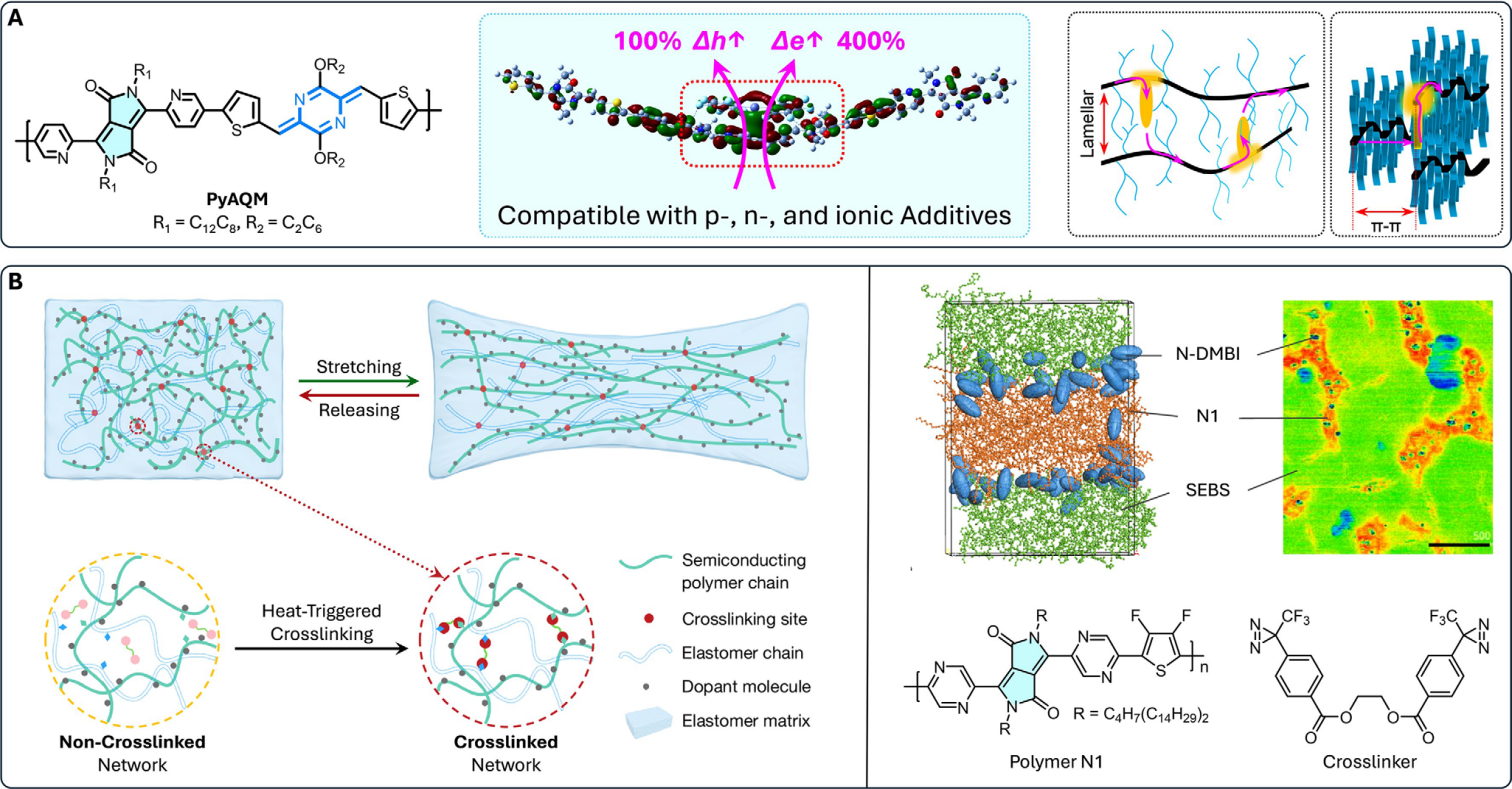
(A) Schematic illustration of a para‐azaquinodimethane‐incorporated polymer, highlighting orbital coupling with additives to enhance charge transport along both the lamellar and π‐stacking directions. Adapted with permission [159]. Copyright 2026, Wiley. (B) Schematic illustration of a thermoelectric elastomer during the stretching‐releasing process and the corresponding thermally activatable cross‐linking mechanism, together with the calculated equilibrium configuration and the corresponding atomic force microscopy coupled with infrared spectroscopy (AFM‐IR) image of the N1/SEBS/N‐DMBI blend. Adapted with permission [1]. Copyright 2025, Nature.

Beyond phenyl flanking groups, the incorporation of nitrogen atoms into six‐membered aromatic flankings to form pyridine units significantly reduces steric hindrance between the flanking group and the DPP core. This structural modification enhances backbone planarity and π–π interactions, thereby enabling applications in organic transistors and thermoelectric devices [155, 156, 157, 158]. In both device classes, the incorporation of additives or dopants plays a decisive role in governing charge transport and overall performance. Recently, Liu et al. demonstrated that the lone‐pair electrons of the pyridinic nitrogen, in conjunction with the introduction of a quinoid para‐azaquinodimethane along the polymer backbone, substantially extend and strengthen orbital coupling between the conjugated polymer and a wide range of additives (Figure 13A) [159]. This enhanced orbital overlap facilitates efficient charge transport along both the lamellar and π‐stacking directions, simultaneously enhancing both electron and hole mobilities up to 400% and 100%, respectively, using single additive, eliminating the need for separate p‐ and n‐type dopants. This is the first reported case of such dual enhancement in conjugated polymer system using individual additive. Notably, this strategy is also broadly compatible with p‐type, n‐type, and ionic additives. By circumventing the intrinsic trade‐offs associated with conventional electron‐transfer‐based p‐ and n‐doping mechanisms, this approach opens new avenues for additive‐ and doping‐assisted performance enhancement in organic semiconductors.

Complementing this molecular‐level strategy, Jang et al. developed a selective doping approach based on solvent affinity and successfully applied it to thermoelectric devices employing a pyridine‐flanked DPP decyanated polymer system [155]. Their study revealed that the affinity between the solvent and polymer backbone, quantified by Hansen solubility parameters (HSPs), critically dictates dopant distribution and the resulting solid‐state morphology. When tetrabutylammonium fluoride (TBAF) was employed as the dopant, solvents exhibiting strong affinity for the DPP polymer disrupted molecular ordering, leading to increased Seebeck coefficients but severely reduced electrical conductivity. In contrast, solvents with intermediate affinity for both the polymer and dopant promoted the formation of mixed‐phase films, in which dopants preferentially accumulated in amorphous regions. Such morphologies preserved high structural order even at elevated doping levels, resulting in electrical conductivities approximately one order of magnitude higher than those achieved using high‐affinity solvents at comparable dopant concentrations. This solvent‐guided doping strategy thus provides an effective framework for precisely tuning electronic properties in doping‐related device processes.

The most recent six‐membered aromatic flanking unit introduced to DPP systems is pyrazine, first reported by Lei et al. [160]. In principle, pyrazine‐flanked DPPs can be synthesized from brominated pyrazine nitriles, however, the high reactivity of bromine in the pyrazine ring often leads to undesired substitution during synthesis. To overcome this challenge, Lei et al. developed an alternative synthetic route involving initial replacement of bromine with an alkoxy triflate (OTf), followed by controlled re‐bromination. This approach enables precise functionalization of the pyrazine unit and has since facilitated the widespread adoption of pyrazine‐flanked DPPs in high‐performance organic transistors and thermoelectric devices [161]. Recently, pyrazine‐flanked DPP polymers have been utilized to fabricate n‐type thermoelectric elastomers (TEEs), representing a significant advance in stretchable energy‐harvesting materials [1]. Unlike stretchable organic field‐effect transistors, where charge transport is largely confined to the semiconductor‐dielectric interface and mechanical recovery is predominantly governed by substrate elasticity, TEEs require uniform bulk charge transport, a considerably more stringent requirement. To overcome this limitation, Liu et al. developed an integrated materials design strategy that combines uniform bulk nanophase separation, thermally activated cross‐linking, and targeted doping within a single polymer‐elastomer system (Figure 13B), yielding TEEs with exceptional rubber‐like elasticity and strain tolerance up to 150%. This design strategy is built upon three key design principles: (i) HSPs were employed to predict and optimize the miscibility between conjugated polymer and the insulating elastomer, enabling uniform nanoscale phase separation; (ii) Thermally activatable cross‐linkers were introduced to reinforce the polymer network, thereby enhancing mechanical robustness; and (iii) Thermally activatable dopants were preferentially diffused into conjugated polymer‐rich domains to enable targeted doping. Molecular dynamics simulations, together with AFM‐IR (atomic force microscopy coupled with infrared spectroscopy) measurements (Figure 13B), confirmed that the dopant N‐DMBI preferentially interacts with the conjugated polymer rather than the elastomer matrix, owing to stronger intermolecular interactions. Upon thermal or photolytic activation, the crosslinker decomposed into reactive carbon species that crosslinked both the aliphatic side chains of the conjugated polymer and the elastomer matrix, effectively suppressing interchain sliding and substantially enhancing elasticity. As a result of these synergistic optimizations, in‐plane thermoelectric devices fabricated from TEEs achieved a maximum power output of 229 nW under a temperature gradient of 48 K. To enable skin‐compatible applications, out‐of‐plane thermoelectric devices were further fabricated, consisting of vertically aligned, free‐standing TEE pillars interconnected by patterned microcracked gold electrodes on a thin SEBS (Styrene‐Ethylene‐Butylene‐Styrene) substrate. When worn on the wrist, these devices generated an output voltage of 2.37 mV. Finite‐element simulations further revealed that, under realistic biomechanical deformation (elbow bending with a radius of 50 mm), the maximum tensile strain experienced by the TEE pillars was only 23%, outperforming conventional organic and inorganic thermoelectric materials. Collectively, these results establish pyrazine‐flanked DPP‐based TEEs as a compelling materials platform that effectively bridges human‐compatible mechanical compliance with efficient energy harvesting, paving the way for next‐generation self‐powered wearable electronics.

## 4 Fused Aromatic Flanked DPP

Compared to five‐ and six‐membered flanking aromatics, fused units promote stronger intermolecular associations between the DPP core and flanking groups [162, 163, 164], thereby enabling enhanced or emerging functionalities. In this context, naphthalene‐flanked DPP (DPPN) represents a prototypical system. Its extended π‐conjugation, well‐defined D‐A architecture, and intrinsic fluorescence provide broad application potential.

**FIGURE 14:**
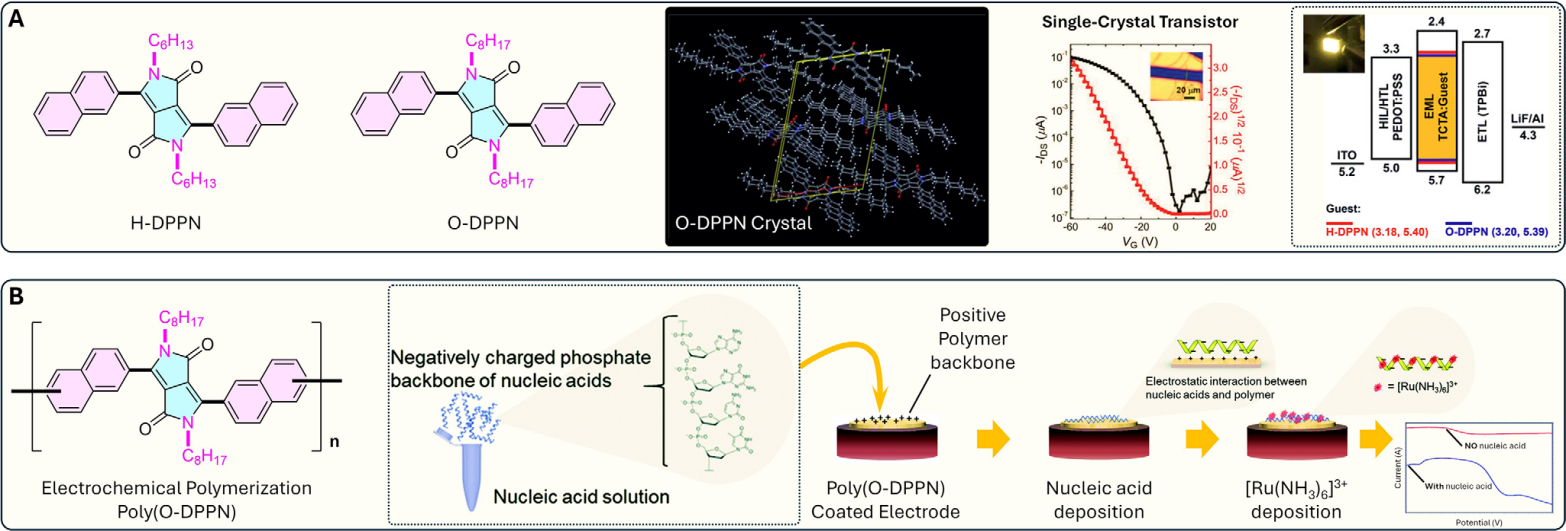
(A) Molecular structures of H‐DPPN and O‐DPPN and their applications in single‐crystal transistors and OLEDs. Adapted with permission [27]. Copyright 2020, Wiley. (B) Electrochemically polymerized O‐DPPN and its application as a biosensor for nucleic acid detection. Adapted with permission [167]. Copyright 2019, RSC.

Liu et al. first reported DPPN monomers demonstrating that DPPN exhibits significantly higher crystallinity compared to its thiophene‐ and furan‐flanked DPP analogues (DPPT and DPPF) [165, 166]. Notably, even as a monomeric semiconductor, DPPN yielded a charge‐carrier mobility five times higher than that of DPPT [165]. Subsequent side‐chain engineering further revealed that linear alkyl substitution simultaneously enhances crystallinity and enlarges crystalline domains [166]. These findings motivated systematic investigations of linearly substituted DPPNs, identifying octyl‐substituted O‐DPPN as the optimal derivative. O‐DPPN achieved a hole mobility of 0.05 cm^2^ V^−1^ s^−1^ in thin‐film transistors, and 0.125 cm^2^ V^−1^ s^−1^ in single‐crystal transistors [27]. In contrast, shortening the side chains to hexyl groups (H‐DPPN) resulted in mobility values nearly an order of magnitude lower. This pronounced performance disparity originates from differences in crystal packing. In H‐DPPN, molecules adopt a chain‐to‐chain stacking motif dominated by offset face‐to‐face π–π interactions between adjacent naphthalene units (3.278 Å). Conversely, as shown in Figures 14A, O‐DPPN forms a stacked molecular backbone with a smaller vertical offset (2.760 Å). These stacks further interact with neighboring chains through naphthalene CH‐π interactions (3.808 Å). Additional CH‐π contacts (2.695 Å) between naphthalene rings and adjacent alkyl chains contribute to a more favorable three‐dimensional packing network, ultimately accounting for the superior charge transport observed in O‐DPPN‐based transistors.

Beyond charge transport, the incorporation of naphthalene unit also imparts DPPN with attractive optoelectronic properties for OLED applications [27]. Devices with the architecture ITO/PEDOT:PSS/TCTA:[H‐DPPN or O‐DPPN]/TPBi/LiF/Al (Figure 14A) exhibited greenish‐yellow emission with CIE (*x, y*) coordinates of (0.50, 0.48). Notably, differences in hole mobility between H‐DPPN and O‐DPPN led to distinct electroluminescence (EL) characteristics. The O‐DPPN‐based device displayed a narrower EL spectrum than its H‐DPPN counterpart, indicating a more confined recombination zone. This behavior arises because, in OLEDs employing TCTA as the host and TPBi as the electron‐transport layer (ETL), charge transport is primarily hole‐dominated, with recombination occurring mainly near the DPPN/ETL interface. The higher hole mobility of O‐DPPN promotes more efficient hole transport toward the ETL, thereby narrowing the recombination zone. In contrast, the lower hole mobility of H‐DPPN results in a broader recombination zone extending toward the hole‐transport‐layer/DPPN interface. Consequently, despite its lower mobility, H‐DPPN‐based OLEDs exhibit higher power efficiency, current efficiency, and external quantum efficiency (EQE) compared to O‐DPPN, likely due to a more balanced charge distribution within the broader zone.

The incorporation of naphthalene moieties introduces additional electroactive sites with relatively low oxidation potentials, facilitating electrochemical polymerization. Meanwhile, the extended π‐conjugation promotes charge delocalization, which is beneficial for the formation of stable polymer films during electrochemical growth. Therefore, H‐DPPN and O‐DPPN have also been investigated as precursors for electrochemical polymerization to yield poly(H‐DPPN) and poly(O‐DPPN) for biosensing [167]. The resulting polymer films exhibited markedly different surface morphologies, which can be traced back to the distinct stacking modes of their respective monomers. Specifically, poly(H‐DPPN) formed a porous structure on glassy carbon electrodes, whereas poly(O‐DPPN) produced smoother films with interconnected ridge‐like features. These distinct morphologies provide effective platforms for biomolecule entrapment. Upon the incorporation of [Ru(NH_3_)_6_]^3+^ into the films, poly(O‐DPPN) exhibited a stronger electrochemical response, particularly during reduction, owing to its smoother morphology. This behavior, combined with the strong redox activity of poly(O‐DPPN), enables its use as a coating for glassy carbon electrodes to interact with nucleic acids via electrostatic attraction. As shown in Figure 14B, nucleic acid deposition onto poly(O‐DPPN) significantly enhances the loading of [Ru(NH_3_)_6_]^3+^, resulting in a pronounced increase in current response. These results clearly demonstrate the promise of poly(O‐DPPN) as a sensitive platform for DNA and RNA biosensing.

**FIGURE 15:**
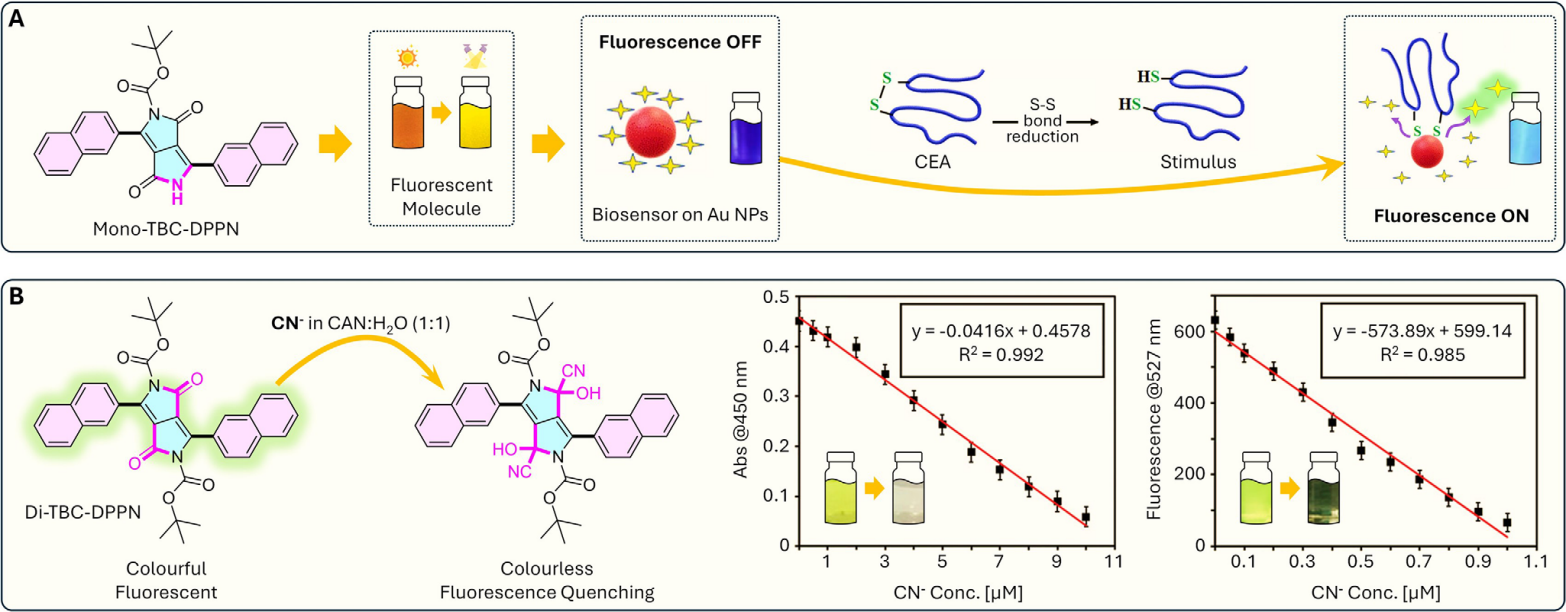
(A) Mono‐TBC‐DPPN and its application as a biosensor for carcinoembryonic antigen (CEA) detection. Adapted with permission [38]. Copyright 2023, Elsevier. (B) Di‐TBC‐DPPN and its mechanical sensing schematic for cyanide ion detection, along with the relationship between absorption/fluorescence responses and cyanide ion concentration. Adapted with permission [168]. Copyright 2021, Wiley.

The incorporation of naphthalene units endows DPPN with strong fluorescence, thereby imparting significant potential for applications in optical biosensing and chemical sensing. To further enable water solubility and improve compatibility with cellular environments or aqueous ion detection, Mahnaz et al. introduced tert‐butyl carbonate (TBC) groups into the material. For instance, Mono‐TBC‐DPPN, which retains free NH groups, was employed to immobilize gold nanoparticles, forming a stimuli‐responsive fluorescent sensor [38]. As illustrated in Figure 15A, the pristine molecule exhibits strong fluorescence that is quenched upon hybridization with gold nanoparticles via plasmon‐induced energy transfer. This platform was successfully applied to the detection of carcinoembryonic antigen (CEA). Specifically, after extracting the protein from human blood plasma, the disulfide bonds in CEA were reduced to sulfhydryl groups. These groups then chemisorbed onto the gold surface, triggering the desorption of Mono‐TBC‐DPPN and the subsequent recovery of its fluorescence emission at 532 nm. This “turn‐on” strategy enabled rapid and sensitive detection of CEA, achieving a limit of detection (LOD) of 10 pg mL^−1^ within 10 min.

In addition to biosensing, a di‐substituted TBC‐DPPN (Di‐TBC‐DPPN) was developed for the selective detection of toxic ions [168]. The sensing mechanism relies on a selective nucleophilic attack by cyanide (CN^−^) ion on the lactam carbonyl groups of the DPP core. This interaction disrupts the rigid π‐conjugated framework, leading to pronounced fluorescence quenching (Figure 15B). This sensing mechanism exhibits high selectivity toward CN^−^ and supports dual‐mode detection through both UV‐Vis absorption and fluorescence spectroscopy in aqueous media. Notably, a distinct colorimetric shift from yellow to colorless allows for naked‐eye detection in acetonitrile/water (1:1, v/v) mixtures. The detection limits were determined to be 5 × 10^−7^ M via UV‐Vis and 5 × 10^−8^ M via fluorescence, both of which meet WHO guidelines for water safety. Supported by density functional theory (DFT) calculations and validated with real samples from contaminated water, this sensor demonstrates strong potential for environmental monitoring and industry safety.

## 5 Fused DPP Architectures

In conventional DPP‐based OSCs, the presence of flanking aromatic units inevitably introduces dihedral torsion. This twisting disrupts backbone planarity, which ultimately limits charge transport and device performance. To address this intrinsic limitation, the direct fusion of flanking units onto the DPP core has emerged as an effective molecular design strategy [169, 170, 171, 172, 173, 174, 175, 176, 177, 178, 179]. By suppressing torsional degrees of freedom, fusion enforces a rigid and highly planar conjugated backbone, thereby enhancing electronic delocalization and intermolecular interactions.

### 5.1 Structure–Property Advantages of Fused DPP

**FIGURE 16:**
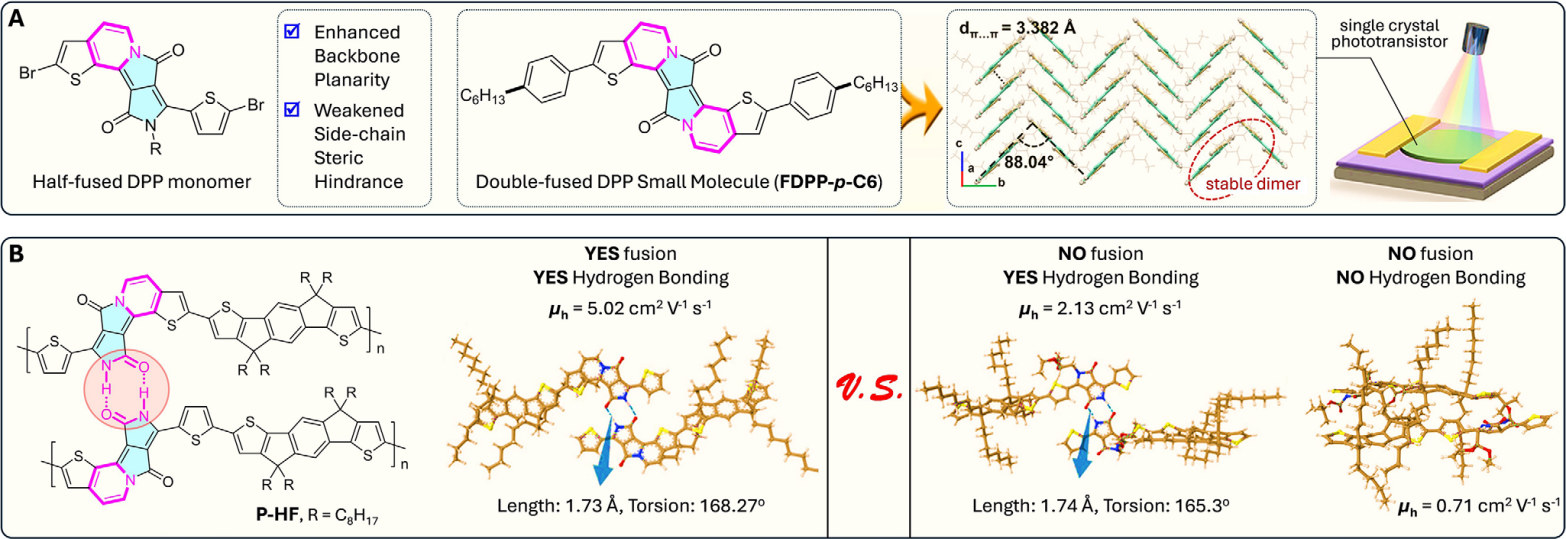
(A) Advantages of half‐fused diketopyrrolopyrrole (DPP) monomer; a double‐fused DPP small molecule and its single crystal for phototransistor application. Adapted with permission [170]. Copyright 2025 Wiley. (B) Combination of fused DPP with hydrogen bonding, and corresponding molecular dynamic simulations compared with control polymers. Adapted with permission [171]. Copyright 2025, Wiley.

Early study on this fusion strategy by Gonka et al. was subsequently extended by Minotto, Shi, and co‐workers to applications in OLEDs and field‐effect transistors [173, 174, 175]. Compared with conventional thiophene‐flanked DPPs, the half‐fused analogue (structure shown in Figure 16A) exhibits markedly improved coplanarity, with dihedral angles below 2°, a significant reduction from the ∼12° observed in nonfused systems. Notably, this near‐planar geometry is preserved across all possible isomers arising from backbone asymmetry. Beyond these geometric benefits, fusion induces an upshift of the HOMO level and a downshift of the LUMO level. This narrow bandgap facilitates balanced hole and electron injection, favoring ambipolar transport. Moreover, fusion alleviates the steric congestion typically caused by side chains, an inherent challenge in conventional DPPs that often necessitates additional structural engineering. The resulting reduction in steric hindrance enables enhanced interchain π–π interactions and denser molecular packing, leading to substantially improved charge transport. For example, a polymer incorporating difluorothiophene into a half‐fused DPP core demonstrated outstanding ambipolar performance with excellent air stability, achieving hole and electron mobilities of 1.08 and 2.23 cm^2^ V^−1^ s^−1^, respectively, under ambient conditions [175].

Chen et al. further advanced this field by synergistically integrating half‐fused DPP units with intermolecular hydrogen bonding. In this specific design, the liberation of the NH group in the half‐fused DPP segment enables the formation of intermolecular hydrogen bonds, which effectively suppresses torsional distortion between the nonfused thiophene and the DPP core [171]. As a result, the dihedral angle is reduced to a negligible value (0.01°), whereas conventional DPP analogues exhibits angles of 3.27° and 30.26°, even when similar hydrogen bonding is present. The resulting polymer (P‐HF, Figure 16B) benefits from a cooperative effect: hydrogen bonding promotes ordered and compact π‐stacking, while the half‐fused core optimizes backbone planarity and energy‐level alignment. Due to this synergy, P‐HF achieves a markedly enhanced hole mobility of 5.02 cm^2^ V^−1^ s^−1^, substantially surpassing polymers incorporating only hydrogen bonding (2.13 cm^2^ V^−1^ s^−1^) or neither strategy (0.71 cm^2^ V^−1^ s^−1^). These compelling results have stimulated growing interest in the fused DPP architecture.

Building upon the success of half‐fused DPP systems, the transition to double‐fused architectures represents a logical progression in molecular design. However, the practical implementation of these systems is often hindered by poor solubility due to their extreme rigidity. To address this challenge, Zhuang et al. introduced flexible alkyl chains onto flanking thiophene units to compare double‐fused DPP‐benzodithiophene motifs with their nonfused analogues [176]. The fused polymers exhibited concurrent enhancements in both hole and electron mobilities. DFT calculations, complemented by electrostatic and van der Waals potential analyses, revealed strengthened intermolecular interactions in the fused systems, which were further corroborated by X‐ray diffraction studies showing markedly improved in‐plane and out‐of‐plane molecular packing.

Relocating the alkyl chains from the flanking thiophene units to the fused segments, as demonstrated by Ma et al., further improved synthetic accessibility and expanded the design space [177]. While the resulting materials displayed ambipolar hole and electron mobilities on the order of 10^−2^–10^−3^ cm^2^ V^−1^ s^−1^, the combination of enhanced processability and simplified synthesis provides a practical route for future optimization through comonomer selection and morphology control. Owing to their rigid, well‐defined frameworks, double‐fused DPPs are also ideal candidate for single‐crystal growth. Cui et al. reported single‐crystal visible‐NIR organic phototransistors based on double‐fused DPP derivatives, where flexible alkyl chains were removed to peripheral sites to minimize steric hindrance (Figure 16A) [170]. Single‐crystal analysis revealed an exceptionally planar backbone and a herringbone packing motif, yielding a hole mobility of 0.20 cm^2^ V^−1^ s^−1^. These phototransistors exhibited a broadband spectral response, high photoresponsivity (2.2 × 10^3^ A W^−1^), and a specific detectivity of 2.8 × 10^10^ Jones, highlighting the potential of fused DPP architecture for high‐performance, light‐driven optoelectronic devices.

### 5.2 Biomedical Applications of Fused DPP

**FIGURE 17:**
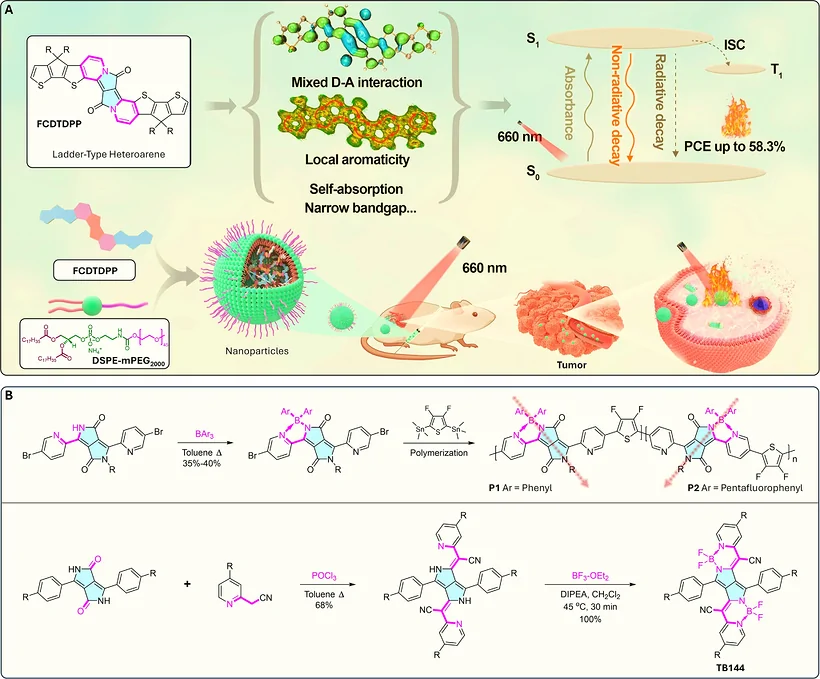
(A) Photothermal conversion efficiency (PCE) of FCDTDPP nanoparticles and the schematic diagram of its photothermal therapeutic application. Adapted with permission [172]. Copyright 2024 Wiley. (B) Facile synthesis of B←N half‐fused pyridine DPP and the pseudoregular polymer.

More recently, the scope of fusion has extended to ladder‐type heteroarenes. Luo et al. constructed a donor–acceptor ladder by fusing a DPP core with cyclopentadithiophene units (FCDTDPP, Figure 17A) [172]. This highly planar architecture facilitates strong intramolecular charge‐transfer (CT) interactions. By modulating the balance between CT and locally excited states, the system achieves intensified, red‐shifted optical absorption. Interestingly, the incorporation of nonaromatic cyclopentadiene units induces localized aromaticity between adjacent cyclopentadiene and thiophene segments, which regulate electron delocalization and excited‐state vibrational dynamics. These features, combined with a narrow bandgap and pronounced self‐absorption, effectively suppress radiative decay pathways, leading to a low fluorescence quantum yield. Consequently, FCDTDPP favors nonradiative decay processes, which are highly advantageous for photothermal conversion. FCDTDPP nanoparticles exhibit an outstanding photothermal conversion efficiency (PCE) of 58.3% under 660 nm irradiation, among the highest performing small‐molecule photothermal agents. Both in vitro and in vivo evaluations have confirmed excellent photothermal imaging capability, biocompatibility, and therapeutic efficacy, underscoring the potential of fused DPP‐based ladder molecules for advanced biomedical applications.

### 5.3 B←N Fused DPP Systems

Beyond thiophene‐based DPP architecture, pyridine has emerged as an attractive flanking unit for the development of n‐type DPP derivatives [137, 180]. Meng et al. reported a half‐fused pyridine‐DPP incorporating a B←N coordination unit, synthesized through a straightforward, high‐yielding route (Figure 17B) [178]. Upon copolymerization with difluorothiophene, the resulting polymer adopts a pseudoregular alternating backbone that promotes predominantly amorphous molecular packing. Such a packing motif is particularly advantageous for flexible electronics, as it ensures mechanical compliance without significantly sacrificing electron mobility. For comparison, a nonfused control polymer proved inactive in flexible transistor configurations employing a PMMA stretchable dielectric layer and a PET substrate. In contrast, flexible transistors based on the fused polymer (P1) exhibited stable electrical performance under repeated bending, along with good ambient stability. These results underscore the promise of fused pyridine‐DPP systems for air‐stable, n‐type applications in wearable sensors and electronic skins. In a related strategy, Thibaut et al. fused DPP units at the lactam (C = O) positions with 2‐pyridylacetonitrile using similar aromatic boron chemistry (TB144, Figure 17B) [179]. Notably, the elimination of the lactam functionalities led to highly transparent materials, enabling their application in transparent dye‐sensitized solar cells. Devices fabricated from these materials achieved a power conversion efficiency of 2.5%, an average visible transmittance of 76%, and a color rendering index of 93, meeting the aesthetic and functional requirements for building‐integrated photovoltaics.

Collectively, these studies demonstrate that judicious selection of fused heteroaromatic cores can impart diverse and application‐specific functionalities. By enforcing planarity and optimizing energy levels, fused DPP architectures have established themselves as versatile platforms for advanced optoelectronic materials. This is particularly evident in the rapid development of fused‐ring dominant fields, such as NFAs, where structural rigidity and precise frontier orbital engineering are paramount.

## 6 Vinyl Bridged DPP (V‐DPP)

Conventional syntheses of aromatic‐flanked DPP derivatives typically rely on aromatic nitrile precursors, which are often costly, synthetically demanding, or inaccessible, particularly for unconventional or halogenated aromatics required for advanced functional materials [181]. This synthetic constraint has historically limited the structural diversity and broader applicability of DPP‐based materials in emerging research areas.

### 6.1 Synthesis and Structural Advantages of V‐DPP

**FIGURE 18:**
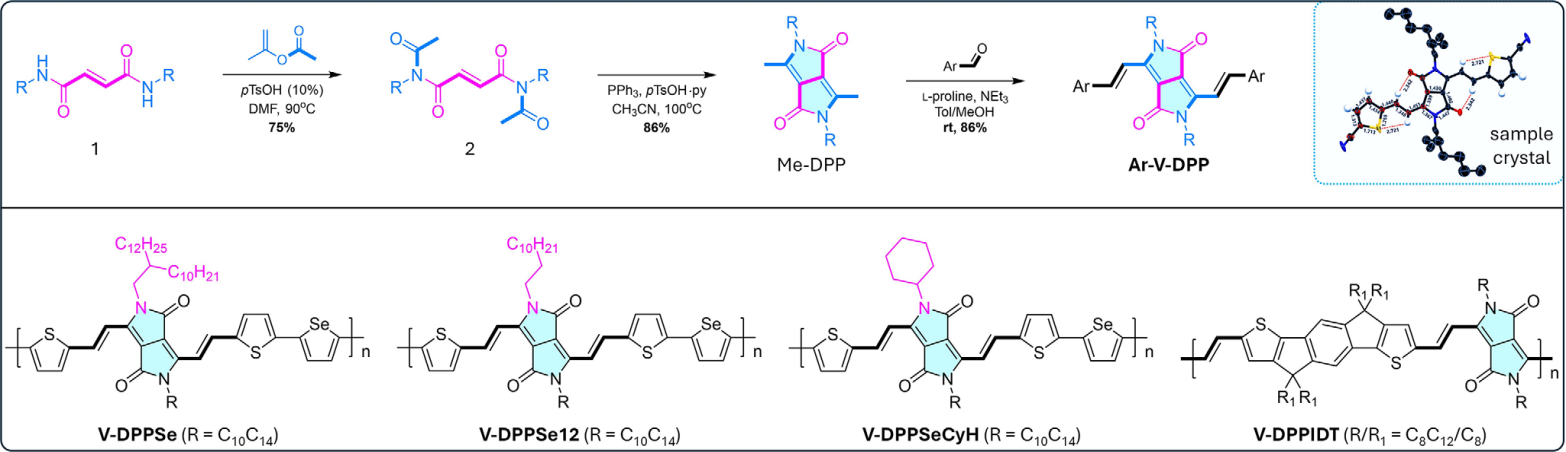
Facile synthesis of vinyl‐linked diketopyrrolopyrrole (V‐DPP), together with a representative single‐crystal structure. Adapted with permission [183]. Copyright 2024, ACS. Chemical structures of the discussed V‐DPP‐based polymers.

To address this challenge, Feng et al. pioneered a versatile synthetic strategy based on vinyl‐linkage formation [182]. As illustrated in Figure 18, methyl‐substituted DPP (Me‐DPP) is first prepared and subsequently subjected to Knoevenagel condensation with a wide range of aromatic aldehydes to afford aromatic‐vinyl‐DPP monomers. This approach dramatically expands the accessible chemical space, as aromatic aldehydes are abundant, inexpensive, and structurally diverse. Importantly, the vinyl bridge not only preserves but also extends the backbone conjugation, while simultaneously reducing steric repulsion between the DPP core and flanking aromatic units. In addition, the overall synthetic yield typically exceeds 50%, a substantial improvement over the ∼10% yields often associated with conventional aromatic‐flanked DPPs. Single‐crystal analysis of a thiophene‐vinyl‐DPP monomer confirms a *trans*‐vinyl configuration and reveals multiple noncovalent interactions, including C–H···O (DPP) and C–H···S (thiophene) hydrogen bonds. These interactions promote one‐dimensional molecular organization with a short π‐π stacking distance of 3.17 Å, effectively shortening donor–acceptor contacts and facilitating charge transport [183]. Collectively, this rational synthetic design enables extensive structural modification and opens a vast design space for emerging applications [181, 182, 183, 184, 185, 186, 187, 188, 189, 190, 191, 192, 193, 194, 195, 196, 197, 198]. Beyond molecular design flexibility, vinyl‐bridged DPP monomers exhibit markedly enhanced chemical reactivity. Chen et al. demonstrated that Stille coupling polymerization of vinyl‐DPP monomers can be conducted under exceptionally mild conditions (60°C for 15 min), yielding polymers with molecular weights exceeding 5 kDa and charge‐carrier mobilities approaching 1.0 cm^2^ V^−1^ s^−1^ [198]. The significantly reduced reaction severity further underscores the synthetic advantages of vinyl‐DPP chemistry for scalable and sustainable material fabrication.

Given that molecular packing and thin‐film morphology are critical determinants of charge transport in conjugated polymers, Chen et al. examined the effects of vinyl incorporation on both parameters. While the insertion of vinyl units between the DPP core and adjacent aromatic moieties effectively reduces backbone steric hindrance, steric effects from side chains remain the primary handle for tuning charge transport [197]. Specifically, the polymer bearing linear side chains (V‐DPPSe12, Figure 18) exhibited minimal steric congestion, achieving hole mobilities as high as 6.76 cm^2^ V^−1^ s^−1^. In contrast, branched side chains in V‐DPPSe introduced additional steric hindrance, resulting in moderate mobilities of ∼3.5 cm^2^ V^−1^ s^−1^, whereas cyclic side chains in V‐DPPSeCyH further increased steric congestion and reduced mobility to 0.83 cm^2^ V^−1^ s^−1^. This pronounced side‐chain‐dependent tunability highlights that targeted modulation of a single structural parameter can effectively optimize charge transport in vinyl‐bridged DPP systems. Beyond side‐chain engineering, Chen et al. further incorporated a large fused indacenodithiophene unit into the DPP backbone via Knoevenagel condensation [194]. Despite exhibiting an amorphous film morphology, the resulting polymer (V‐DPPIDT, Figure 18) maintained a relatively high hole mobility of 1.70 cm^2^ V^−1^ s^−1^ in flexible transistors. As discussed earlier, a central design challenge in stretchable electronics lies in balancing crystallinity and mobility; reduced crystallinity improves mechanical compliance but often compromises mobility. In this context, amorphous polymers that retain high charge‐carrier mobility are highly desirable. These results therefore underscore the promise of vinyl‐bridged DPP materials for future stretchable and flexible electronic applications. Notably, the scalability and mild reaction conditions of Knoevenagel condensation, compared with conventional cross‐coupling reactions, further offer a cost‐effective and environmentally benign route toward large‐area, skin‐like electronic devices.

### 6.2 Optoelectronic Applications of V‐DPP

**FIGURE 19:**
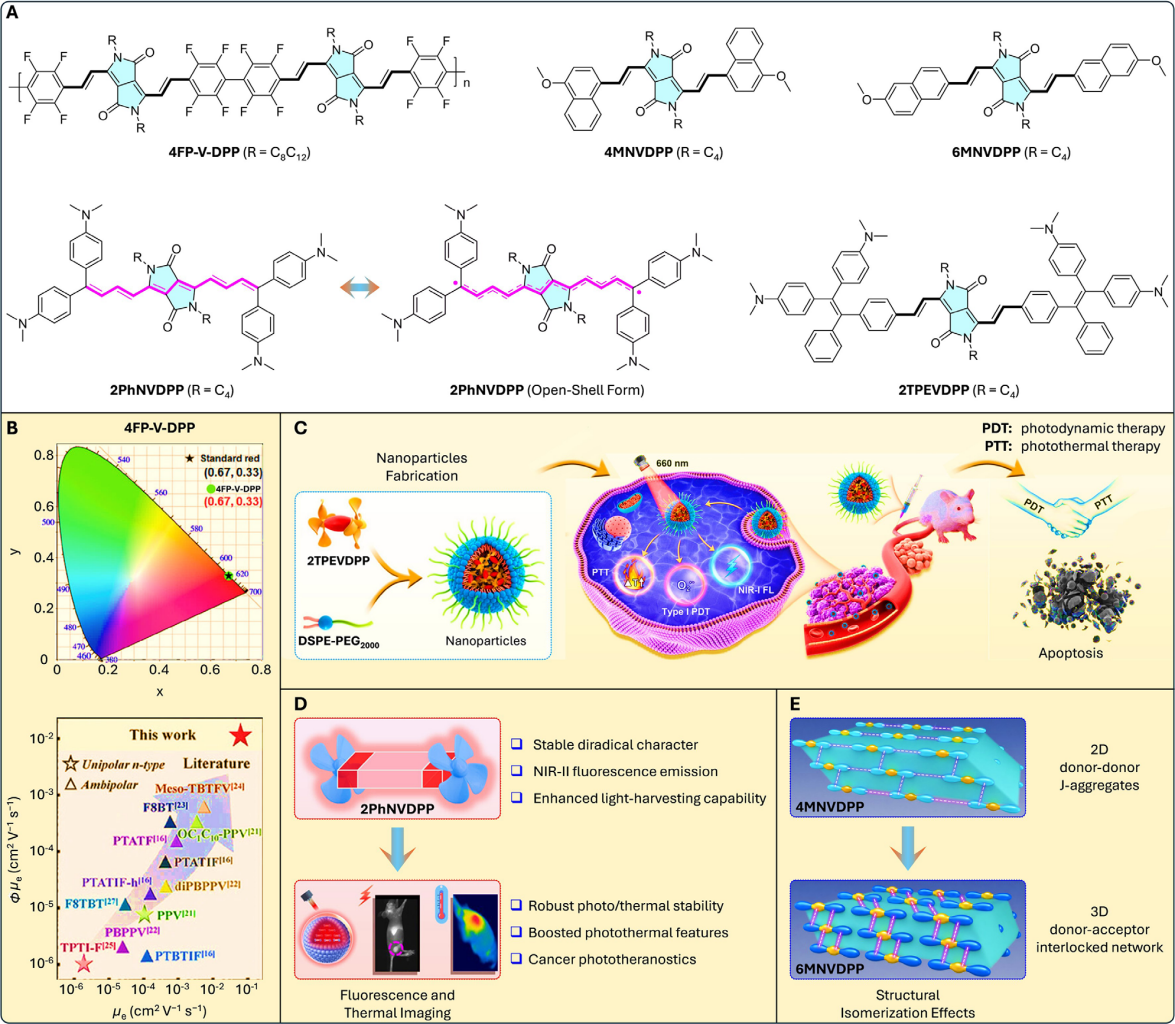
(A) Chemical structures of discussed materials. (B) CIE chromaticity coordinates of 4FP‐V‐DPP and its µ_e_‐Φµ_e_ merit compared with reported materials. Adapted with permission [189]. Copyright 2024, Wiley. (C) Fabrication of 2TPEVDPP nanoparticles and their application for synergistic photodynamic‐photothermal (PDT‐PTT) cancer theranostics. Adapted with permission [187]. Copyright 2022, ACS. (D) Advantages of the stable NIR‐II luminescent diradical 2PhNVDPP for cancer phototheranostics. Adapted with permission [186]. Copyright 2024, ACS. (E) Isomer‐induced variation in molecular packing. Adapted with permission [188]. Copyright 2022, Wiley.

The incorporation of vinyl bridges also extends the optical absorption of DPP‐based materials into the near‐infrared (NIR) region, enabling a range of photorelated applications. Don et al. exploited this extended absorption to enhance the performance of MoS_2_‐based photodetectors [185]. By coating vinyl‐bridged DPPs onto MoS_2_ layer in field‐effect transistors, the photoresponsivity in the NIR region was significantly improved. Specifically, the responsivity at 800 nm increased to ∼10^3^ A W^−1^ (compared to ∼10^2^ A W^−1^ for pristine MoS_2_), while a substantial responsivity of ∼10^2^ A W^−1^ was achieved at 1050 nm where pristine MoS_2_ exhibits a negligible response. These results provide compelling evidence that the optical tunability of organic semiconductors can be harnessed to extend the spectral range of high‐performance inorganic devices. The synthetic versatility of vinyl‐linkage chemistry also permits the incorporation of unconventional aromatic units to optimize charge transport and luminescence simultaneously. In this context, Chen et al. introduced tetrafluorophenyl groups into vinyl‐bridged DPP systems, in which the DPP core primarily governs charge transport while the phenylene‐vinylene segments contribute to luminescence [189]. As a result, the polymer 4FP‐V‐DPP (Figure 19A) exhibits a rare combination of high charge‐carrier mobility and efficient fluorescence. Hole and electron mobilities reached up to 4.6 and 3.7 cm^2^ V^−1^ s^−1^, respectively, while the emission perfectly matches the standard red chromaticity. Such simultaneous realization of high mobility and well‐defined emission remains uncommon among optoelectronic‐integrated conjugated polymers. Notably, the material delivered a maximum Φ·µ_e_ value (the product of fluorescence quantum yield and electron mobility) exceeding 10^−2^ cm^2^ V^−1^ s^−1^, surpassing previously reported emissive semiconductors and outperforming state‐of‐the‐art high‐mobility emissive n‐type polymer semiconductors by several orders of magnitude (Figure 19B). Collectively, these results highlight the effectiveness of vinyl‐linkage engineering in preserving excellent charge transport while enabling multifunctionality.

Another emerging application of vinyl‐bridged DPPs lies in phototherapy, enabled by the incorporation of light‐activated chromophores. Feng et al. demonstrated that vinyl‐bridged DPPs outperform conventional aromatic‐flanked analogues in these applications [187]. In conventional systems, the twisted geometry between the electron‐deficient DPP core and adjacent donor units limit intramolecular donor–acceptor charge transfer, thereby restricting absorption/emission wavelengths and resulting in low PCE. Vinyl‐linked DPPs overcome these limitations by enforcing a highly planar backbone between the DPP core and flanked donor units. This planarity reduces the singlet‐triplet energy gap (Δ*E*_ST_), lowers the optical bandgap, and extends absorption into the NIR‐II region. Meanwhile, bulky donor terminals suppress the detrimental intermolecular π–π stacking that typically leads to fluorescence quenching (aggregation‐caused quenching), while simultaneously enhancing nonradiative decay in the aggregated state. Consequently, the vinyl‐bridged DPP derivative 2TPEVDPP (Figure 19C) functions as a Type I photosensitizer. Unlike conventional Type II systems, Type I photosensitizers are less oxygen‐dependent, making them particularly effective for treating hypoxic tumor environments. Nanoparticles formulated from 2TPEVDPP and DSPE‐PEG2000 exhibited synergistic PDT/PTT, achieving a higher PCE of 66%, significantly higher than the 52% for the aromatic‐flanked analogue. These results indicate that vinyl‐induced planarity between the DPP core and flanking units provides a robust platform with ample flexibility to tune molecular packing, enabling either fully or partially planar backbones to meet device‐specific requirements.

Further extending this concept, researchers discovered that introducing an additional vinyl unit to extend π‐conjugation imparts vinyl‐bridge DPPs with unique radical character. The resulting small molecule 2PhNVDPP adopts a highly planar backbone that facilitates spin delocalization to form radical species. Simultaneously, twisted terminal donor groups effectively suppress excessive π‐π stacking, thereby stabilizing the diradical state (Figure 19D) [186]. Consequently, this molecule exhibits a pronounced spin‐coupling effect and strong NIR light‐harvesting capability. Upon encapsulation into water‐dispersible nanoparticles (NPs), 2PhNVDPP NPs demonstrated exceptional structural stability, favorable biocompatibility, and intrinsic NIR‐II emission with a high PCE of 53%. These integrated properties enabled high‐quality whole‐body vascular and tumor imaging, facilitating successful NIR‐II fluorescence imaging‐guided PTT in vivo. These findings highlight the feasibility of stabilizing robust diradical compounds using vinyl‐bridged DPP architecture, suggesting broader applicability in platforms, such as thermoelectrics, where radical character can be advantageous for enhancing electrical conductivity.

Beyond substituent effects, the isomeric geometry of donor terminals significantly influences molecular packing [188]. A notable example is found in the comparison between linear and bent isomers of naphthalene‐substituted DPP. Specifically, the linear isomer (6MNVDPP, Figure 19E) adopts a three‐dimensional donor–acceptor interlocked network, whereas the bent isomer (4MNVDPP) forms the two‐dimensional donor‐donor‐type J‐aggregates. This distinction arises from strong intermolecular hydrogen bonding between the DPP carbonyl groups and the C–H moieties adjacent to methoxy substituents on the linearly connected naphthalene units. In 6MNVDPP, the hydrogen bond length in the π‐stacking direction is even shorter than the donor–donor noncovalent bond length between naphthalene in 4MNVDPP. As a result, 6MNVDPP exhibits a pronounced red shift of 178 nm in its absorption maximum compared to its bent counterpart, extending deep into the NIR‐II region. This enables 6MNVDPP nanoparticles to achieve outstanding photothermal stability and an exceptionally high PCE of 89%. Collectively, these studies unequivocally demonstrate that the optoelectronic and photophysical properties of vinyl‐bridged DPP materials can be finely tuned through precise molecular and packing engineering. Importantly, the consistent emergence of new structure–property relationships underscores the rich design flexibility of vinyl‐linked DPP systems and highlights substantial opportunities for further exploration across diverse functional applications.

### 6.3 V‐DPP‐Based Covalent Organic Framework

**FIGURE 20:**
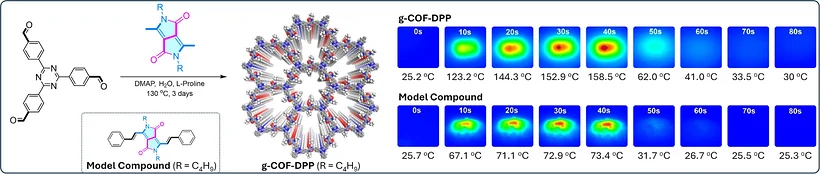
Facile synthesis and stacking mode of g‐COF‐DPP, together with IR thermal images compared with a model compound. Adapted with permission [184]. Copyright 2024, Wiley.

The facile synthesis of vinyl‐bridged DPPs has enabled their successful integration into COFs. Conventional DPP‐based COFs often suffer from limited structural stability due to their reliance on labile imine (C = N) linkages formed via aldehyde‐amine condensation. In contrast, methyl‐substituted DPPs readily undergo Knoevenagel‐type reactions with a broad range of commercially available aldehydes, affording robust vinyl linkages and enabling extensive structural tunability. Several key applications have emerged from this strategy. Vinutha et al. reported triphenylamine‐linked DPP‐COFs for energy storage and thermoelectric applications [192]. In their COF, the tetra‐ene‐like conjugated connectivity promotes resonance between aromatic and radical electronic structures. Upon I_2_ doping, the radical character was significantly enhanced, resulting in a conductivity increase from 10^−3^ to 0.2 S m^−1^. In addition, the electroactive triphenylamine core enabled quasi‐reversible redox behavior. When integrated with graphene oxide, these COFs functioned as an electrode material with a specific capacitance of 70 F g^−1^. Chen et al. employed triphenylphosphine‐based linkers to construct DPP‐COFs that stabilize palladium nanoparticles [195]. The resulting Pd@DPP‐COFs exhibited high catalytic activity in Suzuki–Miyaura cross‐coupling reactions, with excellent stability and recyclability, attributed to the robust framework acting as a durable ligand scaffold. Using a similar three‐dimensional core architecture, Zhang et al. replaced the central N or P atoms with pyridine and triazine units to enhance COF planarity [184]. The electron‐deficient triazine promotes favorable face‐on interlayer stacking, thereby reducing amorphous regions arising from the flexible butyl side chains on DPP. The resulting g‐COF‐DPP exhibited exceptional light‐harvesting capability across the visible‐to‐NIR spectral range and demonstrated stable photothermal responses due to its high crystallinity (Figure 20). These results demonstrate that vinyl‐linkage chemistry constitutes a powerful and versatile strategy for incorporating DPP units into COF architectures, substantially broadening their applicability in energy storage, heterogeneous catalysis, and photothermal energy conversion.

Overall, vinyl‐linkage chemistry represents a powerful and versatile platform for DPP functionalization, enabling unprecedented control over molecular structure, packing, and electronic properties. This strategy significantly broadens the application landscape of DPP‐based materials across organic electronics, optoelectronics, phototherapy, catalysis, energy storage, and photothermal energy conversion, while offering scalable and sustainable synthetic advantages.

## 7 Conclusions and Outlook

To conclude, research on DPP‐based semiconductors has evolved from a primary focus on intrinsic electronic properties toward the exploration of multifunctional characteristics across diverse application scenarios. In this transition, flanking‐group engineering has emerged as a pivotal design lever. On one hand, DPP materials incorporating widely used flanking units, such as thiophene, have been successfully extended into emerging fields including stretchable skin electronics, bioelectronic interfaces, and perovskite photovoltaics. On the other hand, to further broaden the application landscape, increasing attention has been directed toward less conventional flanking motifs that offer a wider and more versatile design space, including nitrogen‐containing heterocycles, fused‐ring systems, DPP‐flanking fused architectures, and vinyl‐bridged strategies.

Against this backdrop, taking flanking‐group engineering as a central design axis, a systematic comparison of five‐membered, six‐membered, fused‐ring, DPP‐flanking fused, and vinyl‐bridged architectures, together with their demonstrated advantages in emerging applications, enables the establishment of a coherent structure–property application relationship across the diverse material systems discussed above. From this perspective, flanking‐group engineering can be understood as operating across three progressively coupled design levels:

Electronic Structure Modulation at the Molecular Level: For widely studied flanking groups (e.g., thiophene, pyridine), their molecular electronic structures and associated structure–property relationships have been extensively elucidated. Consequently, the extension of these materials into emerging applications has largely relied on external strategies, including composite engineering, ternary or block copolymerization, incorporation of biofunctional groups, and device‐level integration.

In contrast, for nonconventional flanking motifs, including fused‐ring systems, DPP‐flanking fused architectures, and vinyl‐bridged DPPs, electronic structure modulation itself serves as the fundamental molecular basis for expanding DPP materials into new application domains. Emerging fusion strategies and vinyl‐bridged designs move beyond simple substitutions to actively reconfigure conjugation pathways and electronic coupling. These unique electronic structures enable, on one hand, the decoupling of traditionally competing properties within a single material system, such as high charge‐carrier mobility with strong luminescence or reduced crystallinity and, on the other hand, significantly broaden the application scope, particularly in areas such as biocompatible PTT, bio/optical sensing, flexible and stretchable electronics, and bioelectronic interfaces. As such, electronic structure regulation via flanking‐group engineering represents a central theme in contemporary DPP research.

Conformational and Packing Control at the Mesoscale: Beyond electronic effects, molecular packing and organization at the mesoscale play a decisive role in determining device performance. Whether extending applications based on conventional flanking systems or optimizing emerging flanking motifs, control over molecular packing remains essential.

A key determinant of packing behavior is molecular conformation. Flanking groups dictate backbone planarity, torsional barriers, and noncovalent interactions, thereby defining molecular conformation and directing mesoscale organization. For example, fused architectures and vinyl‐bridged systems promote planar backbones, suppress conformational disorder, and enhance π‐π stacking, which benefits applications dominated by intermolecular charge transport. In contrast, phenyl‐flanked DPP systems exhibit large torsional angles and twisted backbones, leading to amorphous or porous packing structures that are advantageous for optoelectronic and sensing applications requiring reduced crystallinity or increased free volume.

Furthermore, heteroatom incorporation introduces directional interactions (e.g., S···O, N···H, CH‐π), enabling controlled aggregation, improved domain connectivity, and enhanced morphological stability. These features not only optimize intrinsic material properties but also facilitate multiscale structural control in composite systems, which is critical for applications such as stretchable electronics and bioelectronic interfaces. Notably, vinyl‐bridged systems enable the incorporation of unconventional heteroatom‐rich aromatic units, providing additional degrees of freedom for multiscale packing regulation and thereby accommodating diverse device requirements.

Functional Expansion at the Application Level: Crucially, the molecular‐ and mesoscale‐level features discussed above translate into application‐specific performance through hierarchical organization. As demonstrated across the flanking‐group systems reviewed here, the integration of flanking‐group engineering with complementary strategies enables DPP semiconductors to access an exceptionally broad application space. More importantly, it allows the simultaneous optimization of properties that are traditionally considered mutually exclusive, such as high mobility in amorphous systems, high‐mobility luminescent materials, intrinsically stretchable conjugated semiconductors, thermoelectric materials combining high electrical conductivity and Seebeck coefficient, and biocompatible NIR‐II photothermal therapeutic and sensing platforms. These advances highlight that flanking groups function not merely as structural modifiers, but as functional integrators, enabling DPP‐based materials to meet diverse and often competing device requirements.

By explicitly connecting these three levels, a generalized design framework emerges: flanking‐group selection → electronic structure tuning → molecular packing regulation → device‐relevant functionality. This framework unifies the observations throughout the review into a coherent and transferable set of design principles. Looking forward, both materials design and application expansion will further strengthen and extend this framework.

**FIGURE 21:**
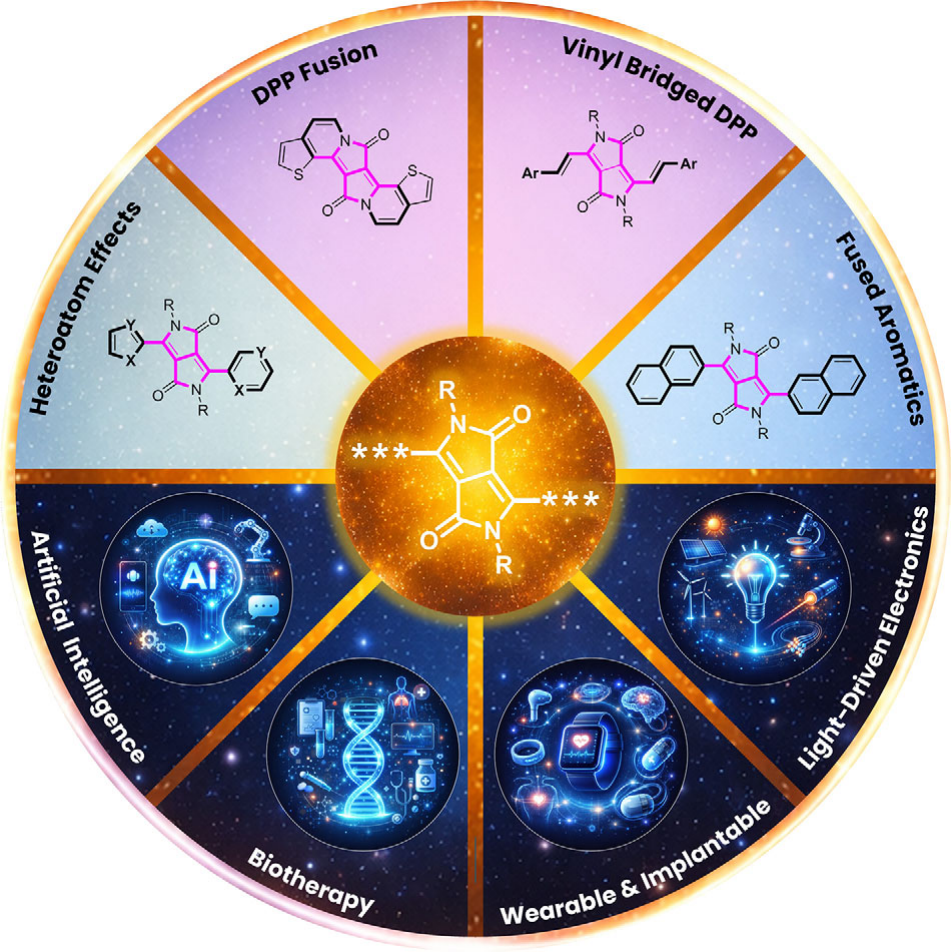
Schematic illustration of future design strategies and application directions of diketopyrrolopyrrole (DPP) based materials. The upper panel highlights emerging molecular engineering approaches, and the lower panel outlines prospective application domains.

From a materials design perspective, greater emphasis should be placed on nonconventional flanking groups, including heteroatom‐rich aromatics, fused architecture, and vinyl‐bridged systems (Figure 21). Although these motifs remain at an early stage, emerging studies have already demonstrated their significant potential, particularly in application‐oriented contexts. Systematic exploration of such systems will not only expand the DPP materials library but also enable the development of more predictive and comprehensive structure‐property relationships.

From an application perspective, DPP‐based materials show strong potential in several emerging areas, including intelligent sensing for artificial intelligence, biocompatible diagnostic and therapeutic platforms, wearable and implantable health‐monitoring systems, and light‐driven electronics (Figure 21). Within these directions, two key challenges remain: the development of environmentally and operationally stable materials and devices, and the realization of multidevice integration, which is essential for translating material‐level advances into practical systems.

Overall, flanking‐group engineering provides a powerful and unifying strategy that transforms the DPP core from a conventional semiconductor motif into a versatile platform for multifunctional materials. By embedding design principles across molecular design, structural organization, and device integration, this approach offers clear and generalizable guidelines for the rational development of next‐generation organic semiconductors.

## Author Contributions

**Xiaoyun Wu**: Writing – original draft, conceptualization. **Shanshan Zhou**: writing – original draft. **Qian Liu**: conceptualization, writing – review and editing, funding acquisition. **Aung Ko Ko Kyaw**: conceptualization, writing – review and editing, funding acquisition. **Xiao‐Lei Shi**: conceptualization, writing – review and editing, funding acquisition. **Meng Li**: writing – original draft.

## Conflicts of Interest

The authors declare no conflict of interest.

## Data Availability

Data sharing not applicable to this article as no datasets were generated or analysed during the current study.
